# Photosensitive Hydrogel with Temperature‐Controlled Reversible Nano‐Apertures for Single‐Cell Protein Analysis

**DOI:** 10.1002/advs.202308569

**Published:** 2024-03-14

**Authors:** Haiyang Xie, Wenke Guo, Hui Jiang, Ting Zhang, Lei Zhao, Jinjuan Hu, Shuxin Gao, Sunfengda Song, Jiasu Xu, Li Xu, Xinyi Sun, Yi Ding, Lai Jiang, Xianting Ding

**Affiliations:** ^1^ Department of Anesthesiology and Surgical Intensive Care Unit Xinhua Hospital School of Medicine and School of Biomedical Engineering Shanghai Jiao Tong University Shanghai 200092 China; ^2^ State Key Laboratory of Oncogenes and Related Genes Institute for Personalized Medicine School of Biomedical Engineering Shanghai Jiao Tong University Shanghai 200030 China

**Keywords:** single cell protein analysis, photoactive polyacrylamide hydrogel, western blot

## Abstract

Single cell western blot (scWB) is one of the most important methods for cellular heterogeneity profiling. However, current scWB based on conventional photoactive polyacrylamide hydrogel material suffers from the tradeoff between in‐gel probing and separation resolution. Here, a highly sensitive temperature‐controlled single‐cell western blotting (tc‐scWB) method is introduced, which is based on a thermo/photo‐dualistic‐sensitive polyacrylamide hydrogel, namely acrylic acid‐functionalized graphene oxide (AFGO) assisted, N‐isopropylacrylamide modified polyacrylamide (ANP) hydrogel. The ANP hydrogel is contracted at high‐temperature to constrain protein band diffusion during microchip electrophoretic separation, while the gel aperture is expanded under low‐temperature for better antibody penetration into the hydrogel. The tc‐scWB method enables the separation and profiling of small‐molecule‐weight proteins with highly crosslinked gel (12% T) in SDS‐PAGE. The tc‐scWB is demonstrated on three metabolic and ER stress‐specific proteins (CHOP, MDH2 and FH) in four pancreatic cell subtypes, revealing the expression of key enzymes in the Krebs cycle is upregulated with enhanced ER stress. It is found that ER stress can regulate crucial enzyme (MDH2 and FH) activities of metabolic cascade in cancer cells, boosting aerobic respiration to attenuate the Warburg effect and promote cell apoptosis. The tc‐scWB is a general toolbox for the analysis of low‐abundance small‐molecular functional proteins at the single‐cell level.

## Graphical abstract

A novel thermo/photo‐sensitive dualistic hydrogel enables constricted pore size at 55 °C to reduce protein diffusion during electrophoresis and an expansion at 4 °C for better antibody penetration during single‐cell immunoblotting. Based on this novel dualistic hydrogel, tc‐scWB provides a universal, highly sensitive, and highly specific platform for low‐abundance small‐molecular protein analysis at single‐cell resolution.

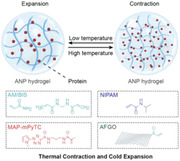

## 1 Introduction

Ubiquitous proteins mediate diverse life activities and perform numerous essential functions in cells and organisms. Important physiological processes such as embryonic development,^[^ ^1^ ^]^ cell differentiation,^[^ ^2^ ^]^ and disease diagnostics^[^ ^3^ ^]^ often involve the simultaneous regulation of multiple proteins across a wide molecular range. The expression level of protein varies in individual cells, and cellular heterogeneity is considered to be closely related to a series of significant processes, including transcription, translation, mRNA, and protein degradation.^[^ ^4^ ^]^ Such subtle cellular heterogeneity may contribute to phenotypic variation^[^ ^5^, ^6^ ^]^ and disease progression.^[^ ^7^ ^]^ However, the mean value of cell population obtained by traditional methods conceals the diverse single‐cell behavior in complex populations. To fully understand the complexity of biological homeostasis and analyze subpopulations of cells with high dynamics and heterogeneity, it is essential to measure protein expression at the single‐cell level.^[^ ^8^ ^]^ However, some functional proteins commonly express with low copies and cannot be amplified like nucleic acids, making quantitative profiling of proteins at single‐cell resolution a general challenge.

Fluorescence flow cytometry is the golden standard approach for multiplex profiling of proteins at the single‐cell level.^[^ ^9^ ^]^ However, commercial flow cytometry instruments are bulky and costly, and have limitations in the measurement of secreted proteins and intracellular proteins. Advances in microfluidics and antibody encoding have made traditional protein quantification technologies into single‐cell multiplexing protein measurements possible.^[^ ^10^ ^]^ Miniaturization of traditional immunoassays, such as western blotting,^[^ ^11^ ^]^ enzyme‐linked immunosorbent assay (ELISA),^[^ ^12^ ^]^ and proximity ligation assay (PLA),^[^ ^13^ ^]^ has developed rapidly in the last decade. To minimize sample volume and thus maximize protein concentration, some microfluidic platforms employed micrometer‐scale chambers to capture individual cells for ultrasensitive detection of the functional proteins at the level of several picograms (pg) of total proteins from single cells.^[^ ^5^, ^14^ ^]^ In addition, antibody‐based immunoassays have a flexible extension mechanism that can encode proteins with metal isotopes or DNA‐conjugated antibodies to further improve the multiplexing and the sensitivity of the method, such as digital proximity ligation assay(dPLA),^[^ ^15^ ^]^ mass cytometry by time of flight(CyTOF),^[^ ^7^, ^16^, ^17^ ^]^ Cellular Indexing of Transcriptomes and Epitopes by Sequencing(CITE‐Seq)^[^ ^18^, ^19^ ^]^ and single‐cell barcode chip(SCBC).^[^ ^20^, ^21^ ^]^ However, antibody‐based methods depend heavily on the antibody binding specificity and might give false positive results in the presence of non‐specific binding.

To improve the detection specificity, single‐cell western blot (scWB), which combines high‐resolution gel electrophoresis and in situ microchip immunoblotting, has been developed for multiplexed profiling of proteins in single cells.^[^ ^22^, ^23^ ^]^ Nevertheless, scWB methods employed the integrally‐formed gel layer, and is still challenging to suppress the diffusion of protein bands in the electrophoretic separation without stacking gel.^[^ ^24^ ^]^ Meanwhile, the joule heat and the electrophoretic separation process would aggravate protein band diffusion, which makes it hard to immobilize proteins onto the gel for subsequent in situ immunoblotting, and may even induce false negative results. In addition, in‐gel antibody probing is practically limited in highly crosslinked gels because of size exclusion, hydrophobic interaction, and electrostatic interactions.^[^ ^25^ ^]^ Therefore, current single‐cell western blot methods are still subject to tradeoffs between in‐gel immunoblotting and separation resolution. An elegant strategy has employed pore‐gradient gel array and a dual cross‐linked polyacrylamide gel (PAG) to achieve optimal single‐cell protein separation in 1 mm long separation lanes.^[^ ^24^ ^]^ The grayscale photopatterning conditions and PAG cross‐linker density need to be optimized in this strategy to support unbiased in‐gel immunoprobing.

**Figure 1:**
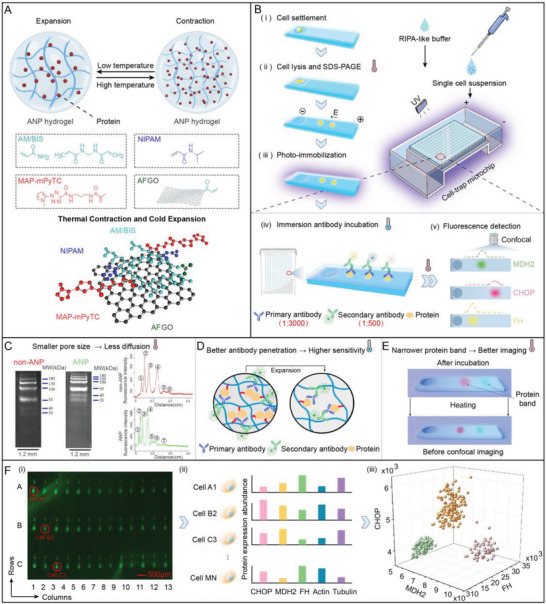
Methodology and Schematics of tc‐scWB based on ANP hydrogel. A) Schematic illustration of photo immobilization of proteins in thermo/photo‐dualistic‐sensitive ANP hydrogel. B) Overview of the tc‐scWB workflow based on ANP hydrogel: i) Single‐cell settlement with gravity. ii) Chemical cell lysis followed by protein electrophoresis. Higher temperature at 55 °C leads to hydrogel contraction, which permits shrinkage of pore size and reduces the spread of protein bands. iii) UV immobilization of proteins in situ inside the ANP hydrogels. iv) Antibody incubation. A lower temperature at 4 °C leads to hydrogel swelling for better antibody immersion and incubation. v) In‐gel fluorescence detection of immobilized proteins. Higher temperature at 55 °C leads to hydrogel re‐contraction and permits a narrower protein signal band. C) Hydrogel contraction allows less protein diffusion during electrophoresis. Visual images and corresponding intensity of purified fluorescently labeled proteins (35–180 kDa) in 10% T non‐ANP and ANP hydrogels. 1) 180 kDa, 2) 130 kDa, 3) 100 kDa, 4) 55 kDa, 5) 40 kDa, 6) 35 kDa, 7) 25 kDa. D) Schematic illustration of hydrogel swelling for better antibody penetration. E) Schematic illustration of hydrogel contraction for a narrower signal band. F) Precise cell subpopulation analysis at the single cell level by tc‐scWB. i) High throughput sensitive immunoblotting bands with position information. ii) High throughput single‐cell multiplex protein profiling with position information. iii) Accurate subpopulation analysis of single cells based on 3D protein expression patterns.

Herein, we introduce a convenient and sensitive single‐cell immunoblotting method, based on a photosensitive hydrogel with temperature‐controlled reversible nano‐apertures, namely acrylic acid‐functionalized graphene oxide (AFGO) assisted, N‐isopropylacrylamide (NIPAM) modified polyacrylamide (ANP) hydrogel. The ANP hydrogel combines synthesized AFGO, (1‐methyl‐1H‐pyrrol‐2‐yl)−2H‐tetrazole‐5‐carboxamide (mPyTC), and NIPAM, possessing an intriguing yet counter‐intuitive property of “heat‐shrinkage and cold‐expansion” (**Figure** **1**A). Acting as both a sieving and probing scaffold, the ANP hydrogel effectively sieves protein targets during SDS‐PAGE, and permits better penetration of the antibodies during probing. The detailed workflow of tc‐scWB is described in Figure 1B. The thermo/photo‐dualistic‐sensitive ANP hydrogel allows the pore size to contract for better electrophoresis separation at high temperature (Figure 1C), to expand for enhanced antibody penetration at low temperature (Figure 1D), and to re‐contract at the imaging step to obtain a narrower signal band again at high temperature (Figure 1E). The tc‐scWB method enables separation and profiling of small molecule weight proteins with highly crosslinked gel (14% T) in SDS‐PAGE, while the in situ immunoblotting signal is enhanced about 16‐fold when compared to conventional scWB method. Therefore, the ANP hydrogel enables scWB to detect low‐abundance and small‐molecular proteins through refined gel composition and a simple temperature‐mediated strategy. Repeated antibody staining and stripping on the same encoding chip allows simultaneous analysis of various target proteins at the single cell level, hence obtaining new insights into cellular heterogeneity under dynamic alternation of cell states (Figure 1F). For the demonstration, we applied tc‐scWB to quantify three low abundant proteins (CHOP, MDH2, and FH) related to tumor metabolic and ER stress in four different pancreatic cell subtypes, namely HPDE, BxPC‐3, Panc1 and SW1990. Single‐cell proteomic profiling reveals the highly dynamic feature of the expression levels of CHOP, MDH2, and FH proteins in different pancreatic cell subtypes. We find FH expression is significantly up‐regulated after enhanced ER stress as triggered by tunicamycin stimulation. We show that the tc‐scWB method can be used to explore the pancreatic cell metabolism status against the expression of ER stress‐specific protein (CHOP) and pivotal enzymes of the Krebs cycle (MDH2 and FH). tc‐scWB can be easily extended to other in situ immunoblotting scenarios for ultrasensitive low‐abundance small‐molecular protein profiling at the single‐cell level.

## 2 Results

### 2.1 Manufacture and Characterization of Thermo/Photo‐Dualistic‐Sensitive Hydrogel

Tetrazole‐based photo click chemistry, as a bioorthogonal reaction, provides promising opportunities for spatiotemporal control of protein imaging in mammalian cells.^[^ ^26^, ^27^ ^]^ The tetrazole ligation is mild and fast, with the unique advantage of a high yield of photoactivated 1,3‐dipolar cycloaddition reaction.^[^ ^28^ ^]^ Inspired by this, we recently developed a tetrazole‐functionalized photoactive polyacrylamide gel,^[^ ^29^, ^30^ ^]^ termed MAP‐mPyTC gel, for highly efficient in situ protein photo‐immobilization in single‐cell western blotting (Figure S1, Supporting Information). Through photo click proximal electrophilic cycloaddition reaction, the carboxylate group of proteins reacts rapidly with carboxy‐nitrile imine intermediates via UV incitation (Figure S2, Supporting Information).

**Figure 2:**
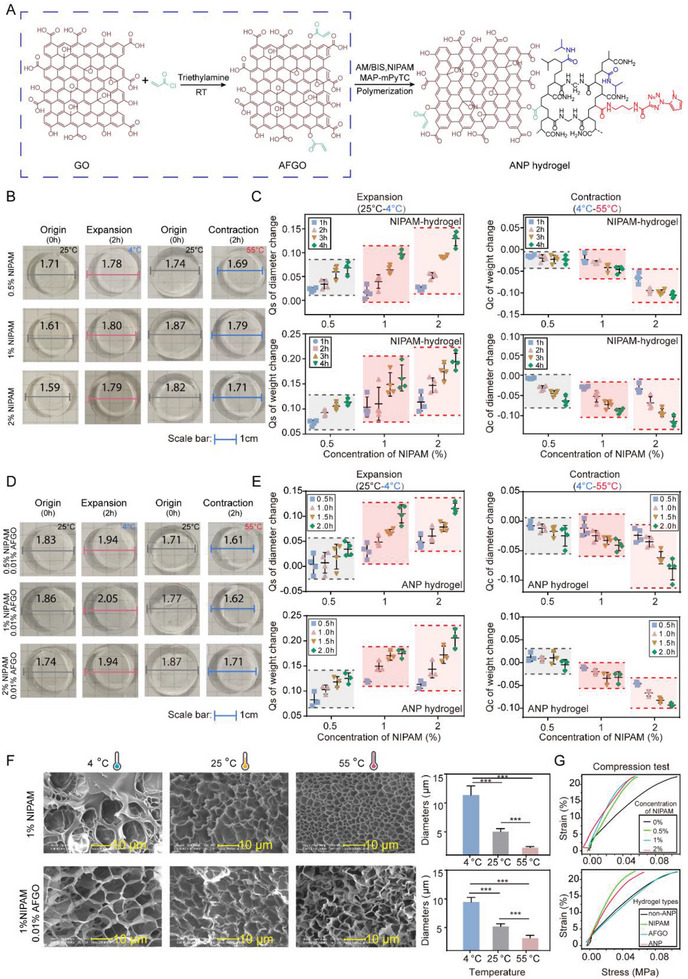
Synthesis of ANP hydrogels and characterization of temperature‐sensitive properties with different NIPAM doping conditions. A) The schematic of ANP hydrogel synthesis. Photosensitive functional groups are marked in red. Temperature‐sensitive functional groups are marked in blue. Acrylic groups are marked in green. B) Optical images of the swelling and shrinking of NIPAM‐hydrogels (0.5%, 1%, and 2% NIPAM) at room temperature (25 °C), lower temperature (4 °C), and higher temperature (55 °C). Digits in images indicate scale in cm. C) Swelling rate at 4 °C and shrinking rate at 55 °C for the same batch of NIPAM‐hydrogels (0.5%, 1%, and 2% NIPAM). Continuous measurements are obtained from 0 to 4 h at 1 h intervals. D) Optical images of the swelling and shrinking of ANP hydrogels (0.5%, 1%, and 2% NIPAM plus an extra 0.01% AFGO) at room temperature (25 °C), lower temperature (4 °C), and higher temperature (55 °C). Digits in images indicate scale in cm. E) Swelling rate at 4 °C and shrinking rate at 55 °C for the same batch of ANP hydrogels (0.5%, 1%, and 2% NIPAM plus extra 0.01% AFGO). Continuous measurements are obtained from 0–2 h at half‐hour intervals. Data are expressed as mean ± SD. F) SEM images of porous structures in NIPAM‐hydrogels (1% NIPAM) and ANP hydrogels (1% NIPAM, 0.01% AFGO). Scale bar: 10 µm. Histograms show the average pore size of hydrogels at 25, 4, and 55 °C. ^*^ *p* < 0.05, ^**^ *p *< 0.01, ^***^ *p* < 0.001, one‐way ANOVA. G) Stress–strain curve in compression mode with different doping conditions. Upper panel, stress–strain curve of NIPAM‐hydrogels (0%, 0.5%, 1%, and 2% NIPAM). Lower panel, stress–strain of NIPAM‐hydrogels (1% NIPAM), AFGO‐hydrogels (0.01% AFGO), ANP hydrogels (1% NIPAM, 0.01% AFGO), and undoped non‐ANP hydrogels.

Poly(N‐isopropylacrylamide) (PNIPAM) is a temperature‐responsive polymer that shows a sharp phase transition in the water around 32 °C, during which the gel changes from a swollen phase at low temperature (below 32 °C) to a collapsed state at high temperature.^[^ ^31^ ^]^ Therefore, we reasoned that doping N‐isopropylacrylamide as a monomer into the polymerization of photoactive polyacrylamide gel may harvest a thermo/photo‐dualistic‐sensitive hydrogel. **Figure** **2**A shows the schematic synthetic route of ANP hydrogel. To verify this hypothesis, we first prepared the NIPAM‐hydrogels with different doping concentrations (0% – 2% NIPAM) (Figure S3 and Table S1, Supporting Information) and assessed the contraction and swelling performance under different controlled temperature conditions (4, 25, and 55 °C) (Figure 2B; Figure S4, Supporting Information). The detailed calculation method of swelling ratio (Q_S_) and contract ratio (Q_C_) is employed to assess the weight and dimensional changes of the hydrogel.^[^ ^32^ ^]^ We observed that the hydrogel expanded under 4 °C within 4 h, and the endpoint Q_S_ values (weight change) were 1.11 (0.5% NIPAM), 1.16 (1% NIPAM), and 1.19 (2% NIPAM), while the endpoint Q_S_ values (diameter change) were 1.07 (0.5% NIPAM), 1.10 (1% NIPAM) and 1.13 (2% NIPAM), respectively (Figure 2C, left panel). We noted that both weight‐based and diameter‐based Q_S_ were raised in tandem with the increasing doping NIPAM concentrations. Meanwhile, under 55 °C, the Q_C_ values (weight change) were 0.98 (0.5% NIPAM), 0.95 (1% NIPAM) and 0.9 (2% NIPAM), while the Q_C_ values (diameter change) were 0.94 (0.5% NIPAM), 0.91 (1% NIPAM) and 0.89 (2% NIPAM), respectively (Figure 2C, right panel). We also observed the weight‐based and diameter‐based Q_C_ values showed similar patterns to those of Q_S_ values. The effects of temperature‐modulated expansion or contraction are more pronounced at higher NIPAM doping concentrations, and the deformation tends to be stable after 4 h. SEM results showed that the pore sizes increased from 2.28 ± 0.19 to 11.37 ± 1.58 µm as the temperature decreased from 55 to 4 °C (Figure 2F, upper panel). In contrast, the non‐NIPAM‐hydrogel did not exhibit a significant temperature‐sensitive response (Figure S5, Supporting Information).

Of note, NIPAM‐hydrogels require a relatively long time to achieve stable endpoint expansion or contraction (up to 12 h). Moreover, when doping is higher than 2% NIPAM, gelation becomes difficult and the hydrogel surface gets viscous and fragile. Therefore, we measured the mechanical properties of NIPAM‐hydrogel with compressive tests and rebound tests. We observed that at the same stress intensity, the strain gradually became larger as the NIPAM doping concentration increased from 0% to 2%. When reached 2% NIPAM doping concentration, the hydrogel was squeezed and crushed. The mechanical measurement indicates that NIPAM doping decreases the elastic modulus of the hydrogels (Figure 2G, upper panel; Figure S6, left panel, Supporting Information). Meanwhile, the rheological test results also indicated that the ANP hydrogel with 1% NIPAM showed fine property of reversible deformation, while the relationship between strain and stress becomes complex in the ANP hydrogel with 2% NIPAM (Table S2, Supporting Information).

Previous investigations reported the combination of graphite oxide (GO), which has excellent thermal and electrical conductivities,^[^ ^33^ ^]^ with poly(N‐isopropylacrylamide) (PNIPAM) hydrogel in applications of drug release, to achieve a negative thermal response or pH response^[^ ^34^ ^]^ To achieve higher tensile strength and elastic modulus of the NIPAM‐hydrogel, we further applied functional graphene oxide (GO) to form the ANP hydrogels. Through reaction with acryloyl chloride under polymerization inhibition of hydroquinone and catalysis of trimethylamine, GO was introduced with acrylic functional groups (Figure 2A, marked in blue dotted box). The synthesized AFGO was then characterized through FT‐IR and UV–Vis (Figure S7, Supporting Information). The characteristic UV–Vis spectrum peak of GO significantly declines after the modification (Figure S7C, Supporting Information), indicating that GO has been successfully functionalized. The AFGO can be uniformly dispersed in N, N‐dimethylformamide (DMF). The AFGO participates in the polymerization of hydrogel monomers, thus AFGO‐hydrogels remain achromatic and transparent, while GO‐hydrogels become brown with obvious impurities (Supplementary Figure S8). The pure GO cannot be uniformly dispersed in N, N‐dimethylformamide (DMF), and thus GO‐hydrogels become brown with obvious impurities in the polymerization of hydrogel monomers. Therefore, we applied functional graphene oxide (AFGO) to form the ANP hydrogels in our work.

The contraction and swelling performance of ANP hydrogel was evaluated at the same temperature as NIPAM‐hydrogels (Figure 2D; Figure S9, Supporting Information). The weights and diameters of the ANP hydrogels were examined at 0.5 h intervals for two consecutive hours. Compared with NIPAM‐hydrogels, the increasing tendency of Q_S_ and Q_C_ values of ANP hydrogels remains the same. However, the response time required to achieve the same Q_S_ and Q_C_ is shortened from 4 to 2 h (Figure 2E), indicating a faster thermal response. Reduction in time consumption may be attributed to the additional thermal and electrical conductivities of GO, based on the sp^2^ hybrid orbital structures and the strong bond between carbon atoms that leads to efficient transfer of phonon.^[^ ^35^ ^]^ The SEM results revealed that the pore sizes of the ANP hydrogel decreased from 9.44 ± 0.8 to 3.19 ± 0.45 µm as the temperature increased from 4 to 55 °C, and AFGO did not affect the hydrogel morphology (Figure 2F, lower panel). Although SEM results were not the real hydrogel size, they indicated differences in thermal response in NIPAM‐gel and ANP‐gel. However, the pore size of ANP showed no discrepancy under different temperature conditions (Figure S10, Supporting Information). From the results of the compressive/rebound test, we observed that the elastic modulus of the ANP hydrogels was higher than that of NIPAM‐hydrogels (Figure 2G; Figure S6, right panel, Supporting Information). The increase in elastic modulus indicates that AFGO doping restores the mechanical properties of the hydrogel affected by NIPAM incorporation.^[^ ^36^ ^]^ Thus, the ANP hydrogel not only has a shorter thermal response time but also exhibits higher mechanical strength compared to standard NIPAM‐hydrogels, making ANP hydrogel more suitable for tough immunoblotting procedures including shaking and washing, as well as swelling and contraction.

Furthermore, we assessed the influence of nanomaterials (AFGO and NIPAM) doping on electrophoresis. Considering nanomaterials doping reduced the mechanical strength of the hydrogel (Figure 2G), 1% NIPAM doping concentration was chosen hereafter to achieve sufficient deformation with little effect on the hydrogel formation. We assessed the electrophoresis properties of the hydrogel using fluorescent‐labeled protein ladders (10–180 kD). After 90 s of electrophoresis, we examined protein bands on non‐ANP hydrogel (hydrogels without AFGO and NIPAM doping) and ANP hydrogel (Figure S11, Supporting Information). The protein bands were visibly separated by both hydrogels in microchip electrophoresis. Meanwhile, AFGO and NIPAM exert a slight influence on the electrophoretic migration distances of proteins in the microchip electrophoresis probably due to the higher applied electric intensity and shorter electrophoresis time. We also evaluated the protein photo capture performance with fluorescein isothiocyanate labeled bovine serum albumin (FITC‐BSA) (Figure S12, Supporting Information). After 30 s of electrophoresis, the FITC‐BSA was then quickly immobilized under 60 s exposure of to ultraviolet light. The fixation efficiency of non‐ANP hydrogels, AFGO‐hydrogels, NIPAM‐hydrogels, and ANP hydrogels was 94.3%, 93.8%, 88.6%, and 90.2%, respectively, and the statistical results showed no significant difference. Therefore, doping NIPAM or AFGO also has little influence on protein immobilization efficiency. Next, we investigated the stability of ANP hydrogel at different temperatures, electrophoresis times, gel concentrations, and electric field intensity. By using BSA‐FITC (25 µg mL^−1^) as model proteins, the electrophoretic migration distance variations in all four experimental conditions were lower than 5%, indicating the stability of the electrophoresis process (Figure S13, Supporting Information).

### 2.2 Sensitive In Situ Microchip Immunoblotting with ANP Hydrogels

**Figure 3:**
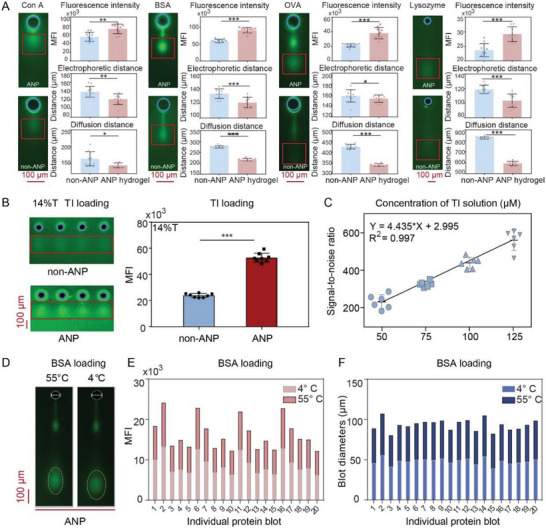
Sensitive in situ microchip immunoblotting with ANP hydrogels. A) Electrophoretic migration behavior visual comparison and the corresponding quantitative data of mean fluorescence intensity, electrophoretic distance, and diffusion distance. Protein loadings from left to right: ConA (104 kDa), BSA (66 kDa), OVA (43 kDa), Lysozyme (14 kDa) with 10% T hydrogel compositions. Electrophoresis time: 30s, scale bar: 100 µm, number of parallel experiments: 10. Data are expressed as mean ± SD. B) Images and corresponding quantitative data of TI immunoblot fluorescence intensity in 14% T non‐ANP and ANP hydrogels. TI loading concentration is 100 µm. Electrophoresis time: 10s, scale bar: 500 µm, number of parallel experiments: 8. C) Fitting curve of signal‐to‐noise ratio to concentrations of TI solution (50, 75, 100, and 125 µm), number of parallel experiments: 6. D) Visual comparison of the same ANP hydrogel immunoblot bands at 4 and 55 °C. FITC‐labeled BSA (1.5 µm) was used as the loading target. Scale bar: 500 µm. E) Comparison of fluorescence intensity of 4 and 55 °C ANP hydrogels. FITC‐BSA loading: 1.5 µm. Before imaging, the microchip is simply dried by the centrifugal force of 2000 rpm. F) Comparison of the blot diameters in 4 and 55 °C ANP hydrogels. FITC‐BSA loading: 1.5 µm. ^*^ *p* < 0.05, ^**^ *p* < 0.01, ^***^ *p* < 0.001(A, C: *t*‐test; D: one‐way ANOVA).

Next, we investigated whether the ANP hydrogel (1% NIPAM, 0.01% AFGO) was applicable for in situ microchip immunoblotting to analyze proteins with different molecular weights. Herein, a high‐temperature (55 °C) electrophoresis buffer was employed to ensure protein denaturation and ANP hydrogel shrinkage. **Figure** **3**A shows the electrophoretic performance of FITC‐labeled Concanavalin A (ConA) (104 kDa), BSA (66 kDa), ovalbumin (OVA) (43 kDa), and Lysozyme (14 kDa) in non‐ANP hydrogel and ANP hydrogel, respectively. When comparing ANP hydrogels with non‐ANP hydrogels, the electrophoretic distance of the four proteins was reduced by 5.9–14.89%, and the diffusion distance was decreased by 13.55–39.16%. Of note, the electrophoretic and diffusion distances of small‐molecular protein (Lysozyme) changed more significantly. Furthermore, under the same protein loading amount, the signal intensity of four different proteins in ANP hydrogels was 18.7–44.38% stronger than that in non‐ANP hydrogels, suggesting ANP hydrogel practically improves the sensitivity of in situ microchip immunoblotting.

Then, we studied the ability of ANP hydrogel for in‐gel immunoblotting at low‐temperature conditions. Conventionally, high concentrations of antibody solution are generally requested for in‐gel immunoblotting due to the steric hindrance and nonspecific adsorption. Herein, we proposed an immersion antibody incubation method to improve the penetration efficiency of antibody molecules into the gel. About 1 mL of diluted antibody solution was injected into each customized incubation bag. The dilution ratio was 1:1000–1:3000. Each ANP hydrogel microchip was placed upward and encapsulated with a heat sealer. These microchips were then placed on a shaker (40–50 r min^−1^) and incubated overnight at 4 °C. After incubation, the antibody solution was collected with a pipette and could be re‐used 2–3 times. Compared with conventional non‐immersed methods (control), the antibody incubation signal was enhanced by 110% for BSA at primary antibody dilution of 1:3000 (0.33 µg mL^−1^) and secondary antibody dilution of 1:500 (1.5 µg mL^−1^) (Figure S14, Supporting Information). The signal enhancement was ascribed to the expanded pore size of ANP gel at 4 °C. Meanwhile, the immersion antibody incubation method requires only 0.67% antibody concentration and allows more than 90% antibody recovery.

The partition coefficient is reduced below 0.2 when hydrogel concentration increases to 12% T,^[^ ^37^ ^]^ making conventional detection of small‐molecular proteins fairly difficult with in‐gel immunoblotting. In previous scWB methods, small‐molecular protein detection requires higher % T hydrogels to attenuate the signal loss due to protein diffusion during the electrophoresis.^[^ ^24^ ^]^ To further assess the performance of in situ immunoblotting with high crosslinking gel, trypsin inhibitor (TI) with a molecular weight of 21 kDa, was employed as a representative of small‐molecular proteins. Compared with non‐ANP hydrogels, the TI blot in ANP hydrogels (14% T) was clearer and showed 120% signal enhancement (Figure 3B). For non‐ANP gels, the fluorescence signal of the antibody at 14% T was hardly detected due to the high cross‐linking. Subsequently, TI solution with different concentration gradients (50–125 µm) was loaded, and the detected signal‐to‐noise ratio (SNR) fit into desired linearity (*R* ^2 ^= 0.997), indicating insignificant deleterious effects on target antigenicity and immunoblotting performances (Figure 3C).

Hydrogel expansion facilitates the antibody incubation but also diminishes the fluorescence signal per unit area. Therefore, we promoted hydrogel re‐contraction with a temperature of 55 °C before confocal scanning to obtain a narrower and sharper imaging band (Figure 3D). To validate the performance of ANP hydrogels, we placed the microchip in a 55 °C water bath and compared the mean fluorescence intensity (MFI) of the same microchip before and after temperature modulation. When the surrounding temperature for hydrogels was switched from 4 to 55 °C, antibody signals increased by 18% ± 13% (Figure 3E). We also observed a 7% ± 5% reduction in blot size (Figure 3F) and a 22% ± 15% reduction in microwell diameters (Figure S15, Supporting Information). Thus, the hydrogel re‐contraction contributes to the enhancement of protein band readout signals.

### 2.3 Sensitive Profiling of Small‐Molecular Weight Proteins in Single Cells Using tc‐scWB

**Figure 4:**
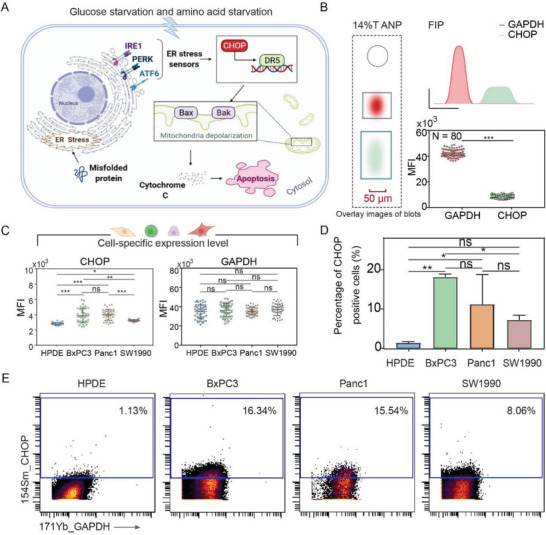
The tc‐scWB unveils highly heterogeneous expression of CHOP protein among pancreatic cell subtypes. A) Stimuli such as glucose starvation and hypoxia can cause unfolded protein response (UPR) and further induce ER stress, which profoundly activates CHOP regulating translation and apoptosis. B) Left panel, overlay images of CHOP and GAPDH fluorescent immunoblotting in a single pancreatic cell with 14% T ANP hydrogels. Right panel, schematic illustrations of fluorescence intensity profile (FIP) and quantitative data of CHOP and GAPDH fluorescent immunoblotting. (Number of cells: 80; Number of parallel experiments: 3) C) CHOP expression heterogeneities in pancreatic cancer cells. Left panel, the MFI (Mean fluorescence intensity) of CHOP. right panel, the MFI of GAPDH. (Number of cells: 100; Number of parallel experiments: 3) D) Quantitative data of CHOP‐positive cells. ^*^ *p* < 0.05, ^**^ *p* < 0.01, ^***^ *p* < 0.001(B: *t*‐test; C, F: one‐way ANOVA). E) The proportion of cells expressing CHOP in BxPC3, Panc1, SW1990, and HPDE cells via mass cytometry analysis.

Next, we investigated the potentiality of ANP hydrogel for sensitive profiling of small‐molecular weight proteins in single cells. Endoplasmic reticulum (ER) stress (**Figure** **4**A) is an essential biological process in carcinogenesis, neurodegeneration, and tumorigenesis.^[^ ^38^ ^]^ C/EBP‐homologous protein (CHOP, 19 kDa) is known as a characteristic marker and a molecular chaperone of ER stress, which regulates translation and apoptosis.^[^ ^39^, ^40^ ^]^ Here, we explored CHOP expression heterogeneities in HPDE pancreatic cells (normal cells) and three types of pancreatic cancer cells, including BxPC‐3, Panc1, and SW1990. tc‐scWB results showed that the expression of CHOP was extremely low both in pancreatic normal cells and cancer cells. The fluorescent signal of internal reference GAPDH was ≈5.5 times stronger than that of CHOP at the single‐cell level (Figure 4B). Moreover, we observed the highly dynamic nature of CHOP expression in all analyzed cells. Taking the HPDE cells with the lowest CHOP expression level as the baseline, the proportion of CHOP‐positive cells in BxPC3, Panc1, and SW1990 cells was 13.1%, 11.2%, and 3.5%, respectively (Figure 4C, left panel). We find CHOP was highly expressed in BxPC3 and Panc1 cells, but presented a general low expression in HPDE and SW1990 cells, while GAPDH expression showed no statistical difference among the four cell subtypes (Figure 4C, right panel). The heterogeneity of CHOP expression in four cell subtypes was further confirmed via mass cytometry, confirming the conclusion robustness of profiling small‐molecular weight proteins by tc‐scWB in single cells. The proportion of CHOP‐positive cells in HPDE, BxPC3, Panc1 and SW1990 cell subtypes was 1.137%, 16.34%, 15.54%, and 8.06%, respectively (Figure 4D,E). The gating strategy shown in Figure S16 (Supporting Information) was used to discriminate single cells, live cells and CHOP‐positive cells.

### 2.4 Profiling of ER Stress and Metabolic‐Related Proteins Using tc‐scWB

**Figure 5:**
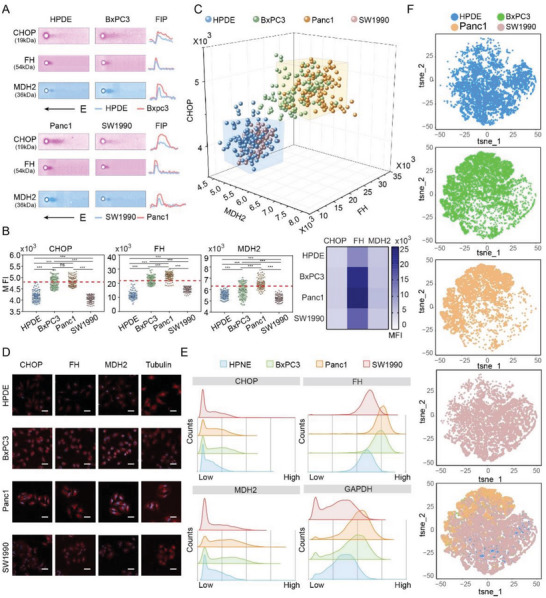
BxPC3 and Panc1 pancreatic cells appear to be more active than SW1990 and HPDE cells in CHOP, MDH2, and FH expression at the basal level. A) Representative single‐cell sensitive immunoblotting fluorescence micrographs and the corresponding fluorescence intensity profile (FIP) of CHOP, MDH2, and FH proteins in HPDE, BxPC3, Panc1, and SW1990 cells. B) Heatmap and statistical comparison of single cell CHOP, MDH2, and FH expression intensity determined via tc‐scWB in HPDE, BxPC3, Panc1, and SW1990 cells (Number of cells: 100; Number of parallel experiments: 3). C) 3D plot of cell distributions according to the expression patterns of CHOP, MDH2, and FH (Number of cells: 400; Number of parallel experiments: 3). D) Expression of CHOP, MDH2, FH, and Tubulin in single HPDE, BxPC3, Panc1 and SW1990 cells via immunofluorescence assay. Scale bar: 50 µm. E) Histograms of CHOP, MDH2 and FH expression in HPDE, BxPC3, Panc1, and SW1990 cells determined by mass cytometry. F) The tSNE plots of expression‐based cell clustering and distribution. Dot points represent individual cells, and the smaller distance between the dots represents the higher similarity of the dots. Color coding represents different cell subtypes. ^*^ *p* < 0.05, ^**^ *p *< 0.01, ^***^ *p* < 0.001, one‐way ANOVA.

We further applied tc‐scWB to investigate the expression of Malate dehydrogenases 2 (MDH2) and fumarase (FH), together with CHOP at the single cell resolution. MDH2 and FH are two pivotal enzymes of the Krebs cycle. MDH2 reversibly converts malate to oxaloacetate, and FH mediates the reversible hydration and dehydration of fumarate to malate.^[^ ^41^ ^]^ Strategies for exploiting metabolic pathways to treat cancer are being actively investigated.^[^ ^42^, ^43^, ^44^ ^]^ We measured the expression of CHOP, MDH2 and FH in four pancreatic cell subtypes. HPDE cells with the lowest CHOP expression were set as the baseline. The proportion of MDH2‐positive BxPC3 cells and Panc1 cells was 31% and 69%, while the proportion of FH‐positive BxPC3 cells and Panc1 cells was 57% and 88%, respectively (**Figure** **5**A,C), indicating higher energy production and altered hyperactive metabolism in cancer cells.

Notably, the MDH2 expression level in SW1990, a cell subtype established from a spleen metastasis of pancreatic adenocarcinoma, was 11.6% and 20.1% lower than that in BxPC3 and Panc1 cells. Similarly, FH expression in SW1990 was 29.9% and 37.1% lower than that in BxPC3 and Panc1 cells (Figure 5B). From the 3D plot, we observed that the expression profile of SW1990 cells was similar to HPDE cells (the normal control cells), while BxPC3 and Panc1 cells exhibit similar protein expression patterns (Figure 5C). SW1990 cells show particularly higher expression of methylenetetrahydrofolate dehydrogenase 2 (MTHFD2). MTHFD2 is an embryo‐ and tumor‐specific folate cycle enzyme. The high expression of MTHFD2 is related to the strengthened anaerobic glycolytic phenotype (Warburg effect) and the downregulation of MDH2 and FH.^[^ ^45^, ^46^, ^47^ ^]^ The mean protein expression levels were further confirmed by immunofluorescence staining (Figure 5D) and mass cytometry analysis (Figure 5E). The distribution pattern of four cell subtypes calculated by the t‐SNE algorithm^[^ ^48^ ^]^ using mass cytometry data for dimensional reduction (Figure 5F) is consistent with the tc‐scWB results. Mass cytometry results further confirmed the metabolic disparities among different types of pancreatic cancer cells, indicating that tc‐scWB could faithfully identify small subgroups within a population of heterogeneous cells.

### 2.5 ER Stress Agonist, Tunicamycin, Upregulated the Aerobic Respiratory‐Related Enzymes in Both SW1990 and HPDE Cells

**Figure 6:**
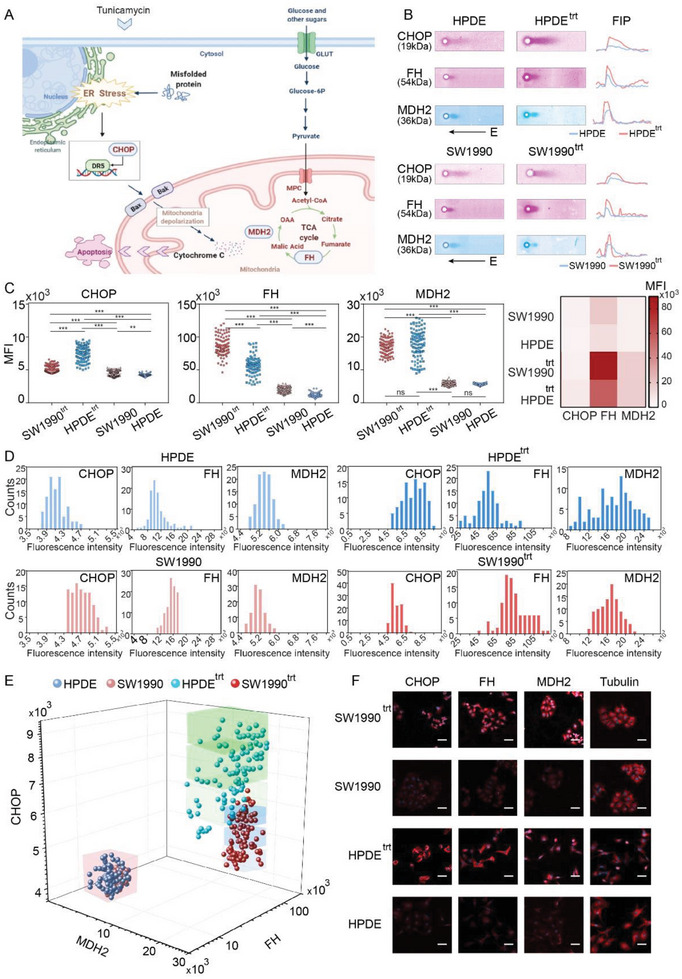
ER stress agonist, tunicamycin, upregulates aerobic respiratory‐related enzymes (MDH2 and FH) expression in both SW1990 and HPDE cells. A) The ANP hydrogel is adopted to decipher the crosstalk between ER stress and mitochondrial metabolism in pancreatic cancer cells. B) Representative fluorescence micrographs and the corresponding fluorescence intensity profile (FIP) of CHOP, MDH2, and FH in HPDE and SW1990 cells before and after tunicamycin treatment. C) Heatmap and statistical comparison of single cell CHOP, MDH2, and FH expression intensity determined via tc‐scWB in HPDE and SW1990 cells before and after tunicamycin treatment. (Number of cells: 100; Number of parallel experiments: 3) D) CHOP, MDH2, and FH expression frequency distribution in HPDE and SW1990 cells before and after tunicamycin treatment. The graph shows the MFI of the total amount of cells examined. E) 3D plot of cell distributions according to the expression patterns of CHOP, MDH2, and FH. (Number of cells: 400; Number of parallel experiments: 3) F) Expression of CHOP, MDH2, FH and Tubulin in single HPDE and SW1990 cells before and after tunicamycin treatment via immunofluorescence assay. Scale bar: 50 µm. ^*^ *p* < 0.05, ^**^ *p* < 0.01, ^***^ *p* < 0.001, one‐way ANOVA.

We then applied tc‐scWB to monitor the effect of ER stress on normal and cancer cell metabolic status with external drug stimulation. We treated HPDE and SW1990 cells with 3 µg mL^−1^ ER stress agonist, tunicamycin, for 6 h. We then adopted tc‐scWB to analyze the expression of CHOP, MDH2, and FH before and after tunicamycin treatment (**Figure** **6**A).^[^ ^49^ ^]^ Figure 6B shows the protein blots of CHOP, MDH2 and FH before and after tunicamycin treatment. CHOP protein expression was significantly upregulated after tunicamycin treatment. In SW1990 cells, CHOP expression was 1.27 times higher than that before stimulation, and in the HPDE cells, CHOP expression increased by 80% (Figure 6C).

The expression of MDH2 and FH was also significantly upregulated after tunicamycin treatment. The expression of MDH2 and FH was increased by 2.22‐fold and 6.3‐fold in HPDE and increased by 2.27 and 4.2 times in SW1990, respectively (Figure 6C). The upregulation of these biomarkers indicates that the intracellular Krebs cycle was enhanced. The upregulation of FH and MDH2 is higher in HPDE than in SW1990 cells, which may be related to the enhanced anaerobic glycolysis phenotype in cancer cells. In addition, frequency distribution indicated that both HPDE and SW1990 cells showed larger heterogeneity after stimulation, and the intercellular heterogeneity in HPDE cells was greater than that in the SW1990 cells (Figure 6D,E). Immunofluorescence results similarly exhibited the upregulation of CHOP, MDH2 and FH protein expressions, which are consistent with the tc‐scWB results (Figure 6F).

When mapped to a 3D plot (Figure 6E), for tunicamycin‐treated cells, we revealed that the expression profile of SW1990 cells was significantly segregated from HPDE cells. Based on the CHOP expression level, we divided SW1990 cells and HPDE cells further into three subpopulations (CHOP^low^, CHOP^middle^ and CHOP^high^). In the CHOP^low^ subpopulation, the FH expression was 35.6% higher in SW1990 cells than that in HPDE cells. The expression of MDH2 in SW1990 cells was 10% lower than that in HPDE cells. Among the CHOP^middle^ subpopulation, FH expression was 33% higher in SW1990 cells than in HPDE cells. The expression of MDH2 in SW1990 cells was 5.4% higher than that in HPDE cells. In the CHOP^high^ subpopulation, FH expression was 34% higher in SW1990 cells than that in HPDE cells. MDH2 expression was 16.2% higher in SW1990 cells than that in HPDE cells. We observed a greater difference in MDH2 expression between HPDE and SW1990 amongst CHOP overexpression cells. The expression level of FH, on the other hand, did not have a significant correlation with CHOP protein expression levels.

## 3 Discussion

The proposed tc‐scWB method has the following features: i) During 55 °C electrophoresis, ANP hydrogel shrinks, which facilitates the separation of a wide molecular range of proteins, and the peak capacity is added in a fixed‐length separation lane (Figure 1C). Besides, the smaller pore size suppresses the diffusion of proteins, which is beneficial for low‐abundance or small‐molecule protein detection in microchip electrophoresis. ii) Under low‐temperature conditions (4 °C), the ANP hydrogel pore size becomes larger and is conducive to antibody probing during in situ immunoblotting. A larger aperture increased the immunoprobe partition coefficient, allowing for a higher local concentration of detection probe, thus improving the sensitivity of protein detection. In addition, large pore size facilitates the adoption of the immersion antibody incubation method and reduces the antibody concentration down to 0.33 µg mL^−1^. Moreover, the tc‐scWB method reduces the background and improves the signal‐to‐noise ratio (SNR). iii) The thermal deformation of ANP hydrogel is reversible. The hydrogel aperture can shrink again to obtain a narrower signal band, further improving the detection sensitivity.

Meanwhile, it should be noted high temperatures may affect antigen‐antibody binding, leading to serious loss of fluorescence signal in tc‐scWB. Therefore, when raising the temperature to shrink the gel before the immunoprobing imaging step, the stability of the antigen‐antibody complex and signal enhancement must be comprehensively considered in the design of temperature control strategies. In addition, although a high‐resolution protein band can be obtained in tc‐scWB compared to the conventional scWB method, the tc‐scWB has more complicated steps and would take a longer analysis time. The sensitivity of tc‐scWB (≈30 000 protein molecules) is approximately the same as the conventional scWB method, because no additional signal amplification strategy is employed. Thus, tc‐scWB is more beneficial for multiplexed protein profiling with small molecule weight in single cells.

The cell metabolic state in different cell subtypes is complex and heterogeneous. Here, we performed a single‐cell level protein analysis of metabolism enzymes FH and MDH2, together with ER stress effector CHOP in four pancreatic cell subtypes. We further explored the quantitative correlation between cellular heterogeneity and the dynamic alternation of cell metabolic states. By characterizing metabolic and ER stress features, the four pancreatic cell subtypes can be divided into two categories. Our results showed that the expression of FH and MDH2 were generally upregulated in cancer cells. Such upregulation may be attributed to heavy proliferation and high energy demand.^[^ ^50^ ^]^ We further measured the expression of CHOP, MDH2, and FH in SW1990 and HPDE cells before and after treatment with tunicamycin, an ER stress agonist. Our experimental results indicated the expression of key enzymes in the Krebs cycle was upregulated after tunicamycin treatment. These observations were further consistently verified through mass cytometry and immunofluorescence. Thus, tc‐scWB is a reliable and convenient platform for single‐cell analysis of intracellular proteins.

Due to the acidic and hypoxic environment within tumors, tumor cells commonly show less dependence on aerobic respiration and tend to undergo more glycolysis.^[^ ^51^ ^]^ The abnormal metabolic circuitry known as aerobic glycolysis (Warburg effect) is increasingly recognized as a hallmark of tumor progression.^[^ ^52^ ^]^ ER stress, as one of the nutritional status sensors, is considered to be closely related to various metabolic pathways. Previous reports indicate that mitochondrial membranes are hyperpolarized in tumor cells.^[^ ^53^ ^]^ ER stress depolarizes the mitochondrial membrane potential. Mitochondrial membrane potential depolarization is unfavorable to the Warburg effect and beneficial to aerobic respiration. Our results revealed that tumor cells escape cell death by adopting glycolysis. ER stress could affect crucial enzyme (MDH2 and FH) activity of metabolic cascade in cancer cells, boosting aerobic respiration and attenuating the Warburg effect to promote cell apoptosis. Unveiling the mechanisms and functions of abnormal mitochondria metabolism and ER dishomoeostasis at the single‐cell level in the future can facilitate etiology research and therapeutic strategies.

## 4 Conclusion

In summary, we developed a temperature‐controlled single‐cell western blotting (tc‐scWB) method based on a newly developed thermo/photo‐dualistic‐sensitive ANP hydrogel. Combined with convenient temperature control strategies, we demonstrated a robust and effective approach to regulate the pore size of the ANP hydrogel for the analysis of functional intracellular proteins with small molecular weight. Moreover, we showed the quantitative correlation between cellular protein heterogeneity and the dynamic alternation of cell metabolic states, which demonstrated tc‐scWB could precisely identify small subgroups within a population of heterogeneous cells. Notably, the ANP hydrogel is compatible with in‐gel immunoblotting methods and can be further applied to other in‐gel detection scenarios, such as in situ nucleic acids sequencing, to facilitate probe penetration and reduce rare sample consumption.

## 5 Experimental Section

### The Synthetic Route of N‐(3‐methacrylamidopropyl)−2‐(1‐methyl‐1H‐pyrrol‐2‐yl)−2H‐tetrazole5‐boxamide (MAP‐myTC)

First, 2‐(1‐methyl‐1H‐pyrrol‐2‐yl)−2H‐tetrazole‐5‐carboxylic acid with N‐(3‐aminopropyl) methacrylamide was synthesized, then N‐(3‐methacrylamidopropyl)−2‐(1‐methyl‐1H‐pyrrol‐2‐yl)−2H‐tetrazole‐5‐carboxamide (MAP‐mPyTC) was synthesized through the EDCl/HOBt catalyzed amide condensation reaction. The detailed synthetic procedures are described in previous work.^[^ ^29^ ^]^

### Preparation of Acrylic Acid‐Functionalized Graphene Oxide (AFGO)

The graphene oxide (GO) was purchased from XFNANO Tech. Co., Ltd (Nanjing, China). The dried GO powder was dissolved in deionized water and the resulting mixture was subjected to ultrasonic treatment for 30 min using a JY99‐II DN ultrasonicator (Ninbo Scientz Biotechnology, China). The GO was thus exfoliated and formed a brown aqueous suspension of GO nanosheets.

The AFGO was synthesized by a reaction of GO, acrylic acid, and dichlorosulfoxide. Briefly, GO dissolved in DMF was subjected to ultrasonication, in an ice bath to prevent high temperature‐induced structure destruction. Acrylic acid, dichlorosulfoxide, hydroquinone (polymerization inhibitor), and triethylamine were then added to the uniformly distributed GO solution. The mixture was stirred in an ice bath and under nitrogen protection for 2 h, followed by incubation at room temperature overnight. The products were purified via repeatedly distilled water rinsing and centrifugation at 10 000 rpm thrice, to remove small‐molecular by‐products. The resulting AFGO was then lyophilized and stored at 4 °C until further use.

### Characterization of GO and AFGO

The morphology of the prepared GO and AFGO was characterized by Fourier‐transform infrared spectrometer (FT‐IR) was used to investigate the functional groups of GO and AFGO. The FT‐IR spectra of samples were obtained in the range of 400–4000 cm^−1^ and the UV–vis spectra were obtained using a spectrometer (Cary 60).

### Swelling Ratio and Contract Ratio

Swelling experiments were performed by immersing dried hydrogels in distilled water at 4 °C for designated hours followed by 55 °C for designated hours. The hydrogels were imaged and the diameters were measured, then weighed after removing the excess water using filter papers. The weight ratio, Q_S1_ = (W_S_‐W_0_)/W_0_, Q_S2_ = (D_S_‐D_0_)/D_0_ and Q_C1_ = (W_C_‐W_0_)/W_0_, Q_C2_ = (D_S_‐D_0_)/D_0_ were used to evaluate the swelling ratio (Q_S_) and contract ratio (Q_C_), respectively. W_S_ is the weight of the swollen hydrogel. W_C_ represents the weight of the contracted hydrogel and W_0_ is the weight of the corresponding original dry hydrogel, D_S_ represents the diameter of the swollen hydrogel. D_C_ is the diameter of the contracted hydrogel and D_0_ is the diameter of the corresponding original dry hydrogel. To investigate the swelling behavior of the modified hydrogels, images and weights were taken at a continuous specific time.

### Morphological Characterization of the Modified Hydrogels

The composition and microstructures of the lyophilized gels under different modification conditions were characterized with a scanning electron microscope (SEM). The as‐synthesized gels were swollen in distilled water at 4 °C or contracted at 55 °C for 3 h. The gels were subjected to liquid nitrogen and then lyophilized using the vacuum drying and freeze drying machine (Ninbo Scientz Biotechnology, China).

### Mechanical Property Measurements

Tensile measurement of the as‐synthesized modified gels was carried out with an Instron 1185 testing machine (USA) at 23 °C. The dimensions of the cylindrical samples are 6.06 mm in diameter and 80 mm in length. The distance between the two clamps is 20 mm. The crosshead speed used for the tensile test is 100 mm min^−1^.

### Microchip Template Preparation

Microarrays of different sizes on the silicon wafers as templates via photolithography were fabricated. To ensure that the hydrogel has the same size arrays, the photolithography procedure was performed according to the protocol provided by the SU8‐3025 manufacturer. For a 60 µm thick template, 3025 photoresists were used at 1250 rpm for coating (30 µm requires higher RPM). The wafer was then baked on a hot plate at 95 °C for 10–30 min. After the soft bake, UV exposure was performed for 5 min. Then post exposure bake (PEB) was performed at 95 °C for 5–10 min. After PEB, the template was rinsed with the SU‐8 developer for 2–3 min cleaned with isopropyl alcohol (IPA) and dried with nitrogen. Finally, the wafers were cured in an oven at 150–200 °C to further cross‐link the material. The finished silicon wafer was stored upright in a cool, dry environment for later use.

### Synthesis of the ANP Hydrogel Microchip

The hydrogel microchip was synthesized by free radical polymerization. Treat slides with silanization reagent for 30 min, then wash twice with methanol, deionized water, and dry with nitrogen. The ANP hydrogel solution was prepared from 60% acrylamide and 9.58x bis(acrylamide), NIPAM, Tris‐HCl, SDS, Tritonx‐100, and ddH_2_O were added. APS and TEMED were added to initiate the polymerization (Table S3, Supporting Information). The precursor solution was dropped onto the silicon wafer template and then covered with the silanized glass slide. The system was left to stand for 20 min. The slides were then lifted from the mold and stored in a wet box at 4 °C until use.

### Cell Culture

The HPDE, Panc1, SW1990, and BxPC‐3 cell lines were obtained from the Chinese Academy of Sciences. Cells were cultured in their corresponding medium supplemented with 10% bovine serum (S601S‐500, Sera Pro) and penicillin/streptomycin (15240062, Gibco): DMEM (SH30243.01, Cytiva) for HPDE, Panc1 and SW1990, RPMI‐1640 (11875093, Gibco) for BxPC‐3 cells. Cells at ≈80% confluence were passaged or collected for subsequent experiments.

### Cell Stimulation

ER stress was induced in cells by treatment with ER stress agonist tunicamycin (3 µg mL^−1^) at the indicated time points: cells were cultured in DMEM medium with 100 mL L^−1^ inactivated fetal bovine serum and grown at 37 °C with 50 mL L^−1^ CO_2_. Log phase cells were divided into experimental and control groups. The cells in the experimental group were treated with 3 ug mL^−1^ of TM for 6 h, and the control group was supplemented with the same volume of culture medium. The cells were collected by centrifugation at the corresponding time.

### Mass Cytometry Analysis

Cells were detached, collected, and stained with 5 µm Cisplatin (Sigma–Aldrich) for 5 min in a 37 °C water bath, and fixed using 1.6% Paraformaldehyde for 10 min at room temperature. Cells were then incubated for 10 min with blocking solution (BioLegend) and stained with a mixed antibody cocktail at room temperature for 30 min, followed by twice PBS washing. The antibodies are listed in Table S4 (Supporting Information). Metal‐labeled antibodies were labeled in‐house using the MaxPar X8 labeling kit according to the manufacturer's instructions (Fluidigm). Mass cytometry data were obtained using Helios (Fluidigm) as previously reported. Briefly, stained cells were suspended in deionized H_2_O, and added 4‐element normalization beads (Fluidigm) immediately before applying the samples to the instrument. Cells were acquired at a rate of 300–500 events s^−1^.

### Immunofluorescence Staining

To assess the expression of CHOP, MDH2, and FH expression in situ, immunofluorescence staining was performed. HPDE, BxPC3, Panc1, and SW1990 cells were seeded to 80% confluence on a cover glass. After removing the culture medium, the cells were washed with PBS thrice, fixed in prewarmed 4% PFA for 20 min, and then stained with corresponding antibodies and secondary fluorescent antibodies. The cell nucleus was further counterstained with DAPI. Fluorescence images were taken by a confocal fluorescence microscope (Zeiss LSM 880) in the corresponding fluorescence channel. The mean fluorescence intensity of individual cells was determined using ImageJ software.

### Temperature‐Controlled Single‐Cell Western Blotting

The experimental procedure of single‐cell western blotting was detailed as follows. About 1 mL of single cell suspension (the cell concentration was 10^4^–10^5^ cells mL^−1^) was added onto the surface of the hydrogel chip and allowed to stand for 10 min. The cells fell into microwells via passive gravity sedimentation. To achieve high throughput results, the cell gravity settling process should be monitored by bright‐field microscopy until single‐cell occupancies of ≈60% were achieved. Also, the cells that do not fall into the wells could be recycled for a second‐round loading to enhance the single‐cell occupation ratio. Then, the excess cells on the hydrogel chip were gently washed away with PBS. At last, the hydrogel chip would be placed in an electrophoresis tank for subsequent scWB analysis. Lysis solution was poured in to chemically lyse the cells in each microwell, releasing proteins; an electric field (40 V cm^−1^) was applied on both sides to perform single‐cell WB for 30 s; the microchips are placed 15 cm below the UV light source (CL‐1000 M, AnalytikJena) and exposed to UV light for 60 s to immobilize proteins in situ via a photosensitizer. The buffer solution used in scWB was a dual‐function lysis/electrophoresis buffer with a final concentration of 0.5 × Tris‐glycine, and the pH of 8.3 is maintained throughout the experiment. The gels were then swelled at 4 °C for immersed antibody incubation, followed by hydrogel contraction at 55 °C for in‐gel fluorescence detection of immobilized proteins.

### Data Analysis

Fluorescence images were collected by Zeiss LSM 880 confocal microscope, a customized MATLAB script (R2018a, MathWorks) was employed for data quantification and analysis, and ImageJ software was adopted for background subtraction. To obtain the mean fluorescence intensity (MFI) of each blot, several steps were performed as follows: i) Select out regions of interest from the original images (ROI; every individual ROI image corresponds to one lane of the immunoblotting image at single‐cell resolution); ii) Background subtraction is performed by subtracting the average background intensity across the entire ROI; iii) Sum the intensities on the separation axis of the ROI and plot the intensity along the separation axis; iv) Gaussian fitting and quality control.

### Statistics

Statistical analysis was performed using GraphPad Prism 7.0. Unpaired Student's *t*‐test was carried out when comparing two groups and paired Student's *t*‐test was performed for self‐comparisons. For multi‐comparison, one‐way ANOVA was utilized to determine the statistical differences, with the significance level set at *p* < 0.05.

## Conflict of Interest

The authors declare no conflict of interest.

## Author Contributions

H.X., W.G., T.Z., and H.J. contributed equally to this work. H.X., W.G., T.Z., H.J., and X.D. were involved in the conception, design, and drafting of the manuscript. H.X., W.G., T.Z., H.J., J.X., H.L., Y.Y., S.H., S.S., L.X., X.D., X.S., and Y.D. performed experiments. H.X., W.G., T.Z., H.J., and X.D. were involved in the editing and revision of the manuscript. X.D. supervised the project, and all the authors read and approved the manuscript.

## Supporting information

Supporting Information

## Data Availability

The data that support the findings of this study are available from the corresponding author upon reasonable request.
