# Supplementary material for: Photosensitive Hydrogel with Temperature‐Controlled Reversible Nano‐Apertures for Single‐Cell Protein Analysis
Source: Adv Sci (Weinh). 2024 Mar 14;11(19):2308569. doi: 10.1002/advs.202308569 (PMC11109651; doi:10.1002/advs.202308569)
Supplement: Supplementary file 1 — Supporting Information [file ADVS-11-2308569-s001.pdf]

ADVANCED SCIENCE
Open Access

Supporting Information

for *Adv. Sci.*, DOI 10.1002/advs.202308569

Photosensitive Hydrogel with Temperature-Controlled Reversible Nano-Apertures for Single-Cell Protein Analysis

*Haiyang Xie, Wenke Guo, Hui Jiang, Ting Zhang, Lei Zhao, Jinjuan Hu, Shuxin Gao, Sunfengda Song, Jiasu Xu, Li Xu, Xinyi Sun, Yi Ding, Lai Jiang and Xianting Ding**

Supplementary Information

Photosensitive hydrogel with temperature-controlled reversible nano-apertures for single-cell protein analysis

Haiyang Xie [a,b †], Wenke Guo [a,b †], Hui Jiang [a,b †], Ting Zhang [a,b †], Lei Zhao [a,b], Jinjuan Hu [a,b], Shuxin Gao [a,b], Sunfengda Song [a,b], Jiasu Xu [a,b], Li Xu [a,b], Xinyi Sun [a,b], Yi Ding [a,b], Lai Jiang [a,b] and Xianting Ding [a,b *]

[a] Department of Anesthesiology and Surgical Intensive Care Unit, Xinhua Hospital, School of Medicine and School of Biomedical Engineering, Shanghai Jiao Tong University, Shanghai 200092, China

[b] State Key Laboratory of Oncogenes and Related Genes, Institute for Personalized Medicine, School of Biomedical Engineering, Shanghai Jiao Tong University, Shanghai 200030, China

† Equal contributing authors

* Correspondence author: Xianting Ding, Ph.D.; Email: dingxianting@sjtu.edu.cn

Figure S1. The synthetic route of N-(3-methacrylamidopropyl)-2-(1-methyl-1H-pyrrol-2-yl)-2H-tetrazole5-boxamide (MAP-myTC)

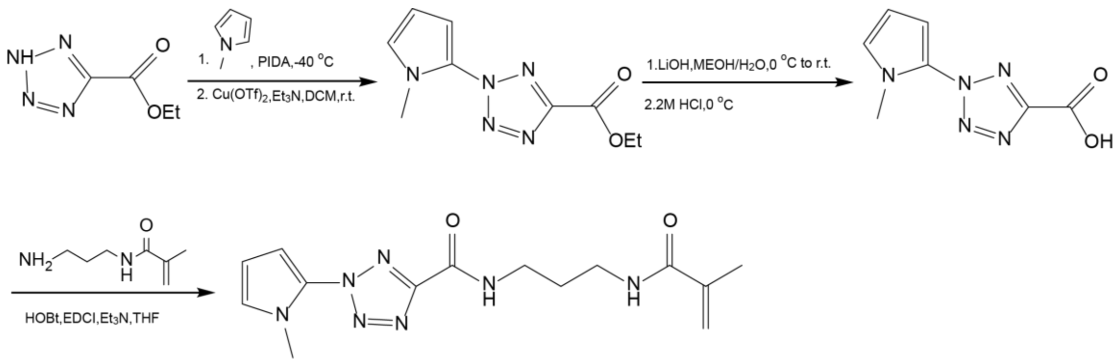

Figure S1. The synthetic route of N-(3-methacrylamidopropyl)-2-(1-methyl-1H-pyrrol-2-yl)-2H-tetrazole5-boxamide (MAP-myTC).

Figure S2. MAP-mPyTC immobilizes the protein in situ with UV excitation

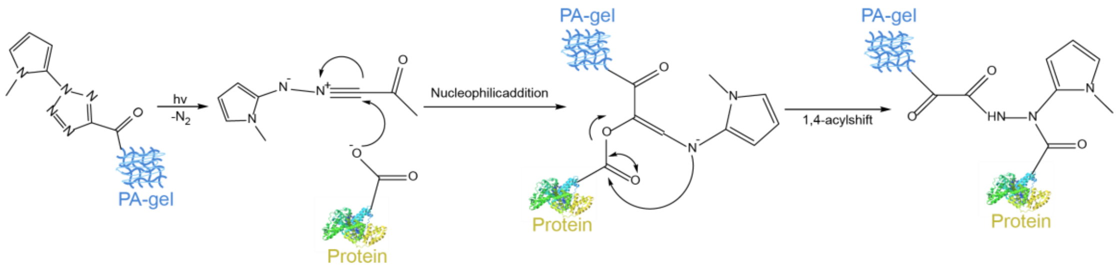

Figure S2. MAP-mPyTC immobilizes the protein in situ with UV excitation. Through classic photoclick proximal electrophilic cycloaddition reaction, the carboxylate group of proteins reacts rapidly with carboxy-nitrile imine intermediates generated via UV incitation.

Figure S3. The synthesis scheme of NIPAM-hydrogels

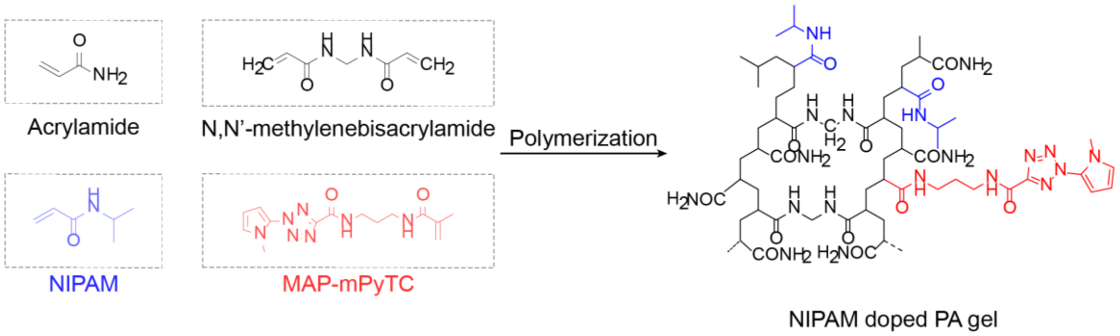

Figure S3. The synthesis scheme of NIPAM-hydrogels. Photosensitive groups are marked in red, and temperature-sensitive functional groups are marked in blue.

Figure S4. Contraction and swelling performance of the NIPAM-hydrogels

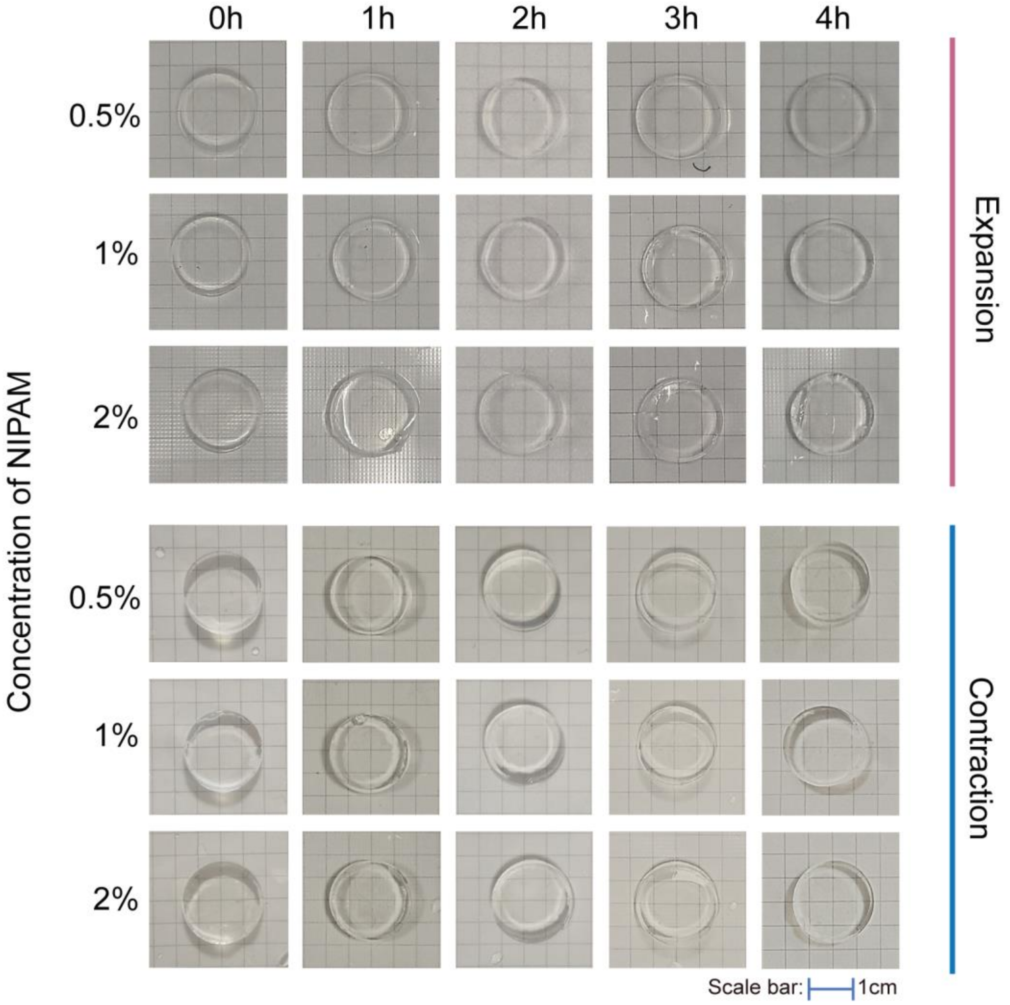

Figure S4. Contraction and swelling performance of the NIPAM-hydrogels (0.5%, 1%, and 2% NIPAM). Optical images of the contraction and swelling performance of NIPAM-hydrogel with different doping concentrations (0.5%-2%) under different controlled temperature conditions, continuous measurements from 0-4 hours at 1h intervals. During the duration of 4 h, the gels expand slowly under 4 °C. While under the temperature condition of 55 °C, the gels contract. The effects of temperature-modulated expansion or contraction are more pronounced at higher NIPAM doping concentrations.

Figure S5. Morphological characterization of the undoped non-ANP hydrogels

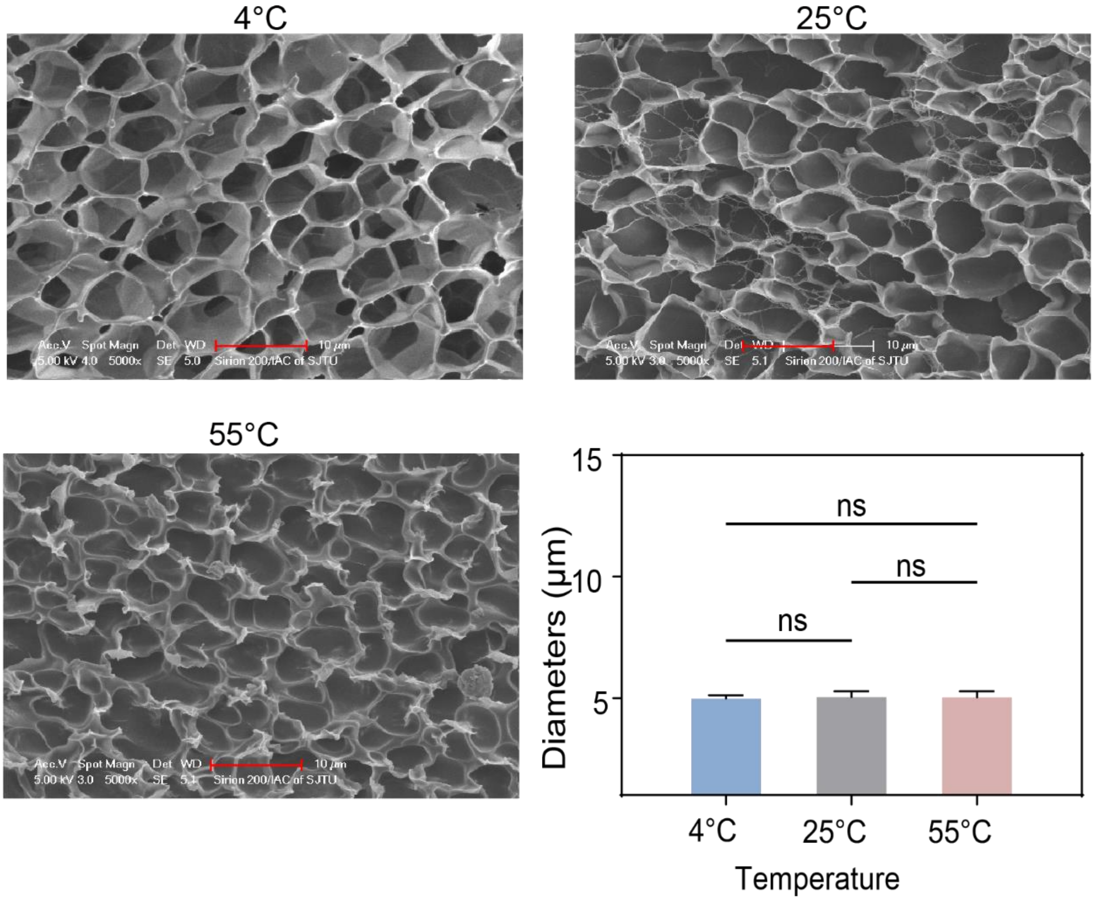

Figure S5. Morphological characterization of the 14T% undoped non-ANP hydrogels. SEM images of porous structures in 14T% non-ANP hydrogels at 25 °C, 4 °C, and 55 °C. Scale bar: 10 μm. Histograms show the average pore size of gels at 25 °C, 4°C, and 55 °C. Data are expressed as mean ± SD. *P<0.05, **P<0.01, ***P<0.001, one-way ANOVA.

Figure S6. Mechanical performance characterization of hydrogels with different doping conditions

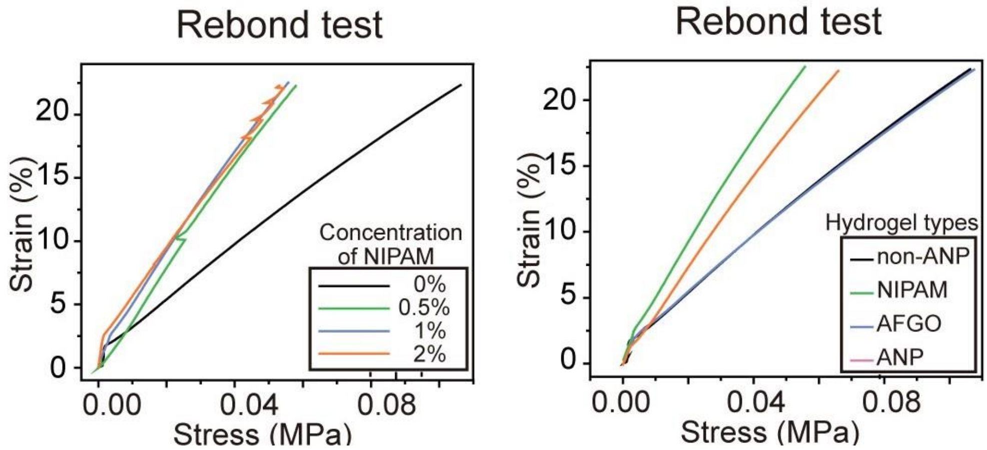

Figure S6. Mechanical performance characterization of PA-gels with different doping conditions. Left panel, stress-strain curve of NIPAM-hydrogels (0%, 0.5%, 1%, and 2% NIPAM) in tensile mode. Right panel, stress-strain of NIPAM-hydrogels (1% NIPAM), AFGO-hydrogels (0.01% AFGO), ANP hydrogels (1% NIPAM, 0.01% AFGO), and undoped non-ANP hydrogels (control) in tensile mode.

Figure S7. Characterization of the synthesized AFGO

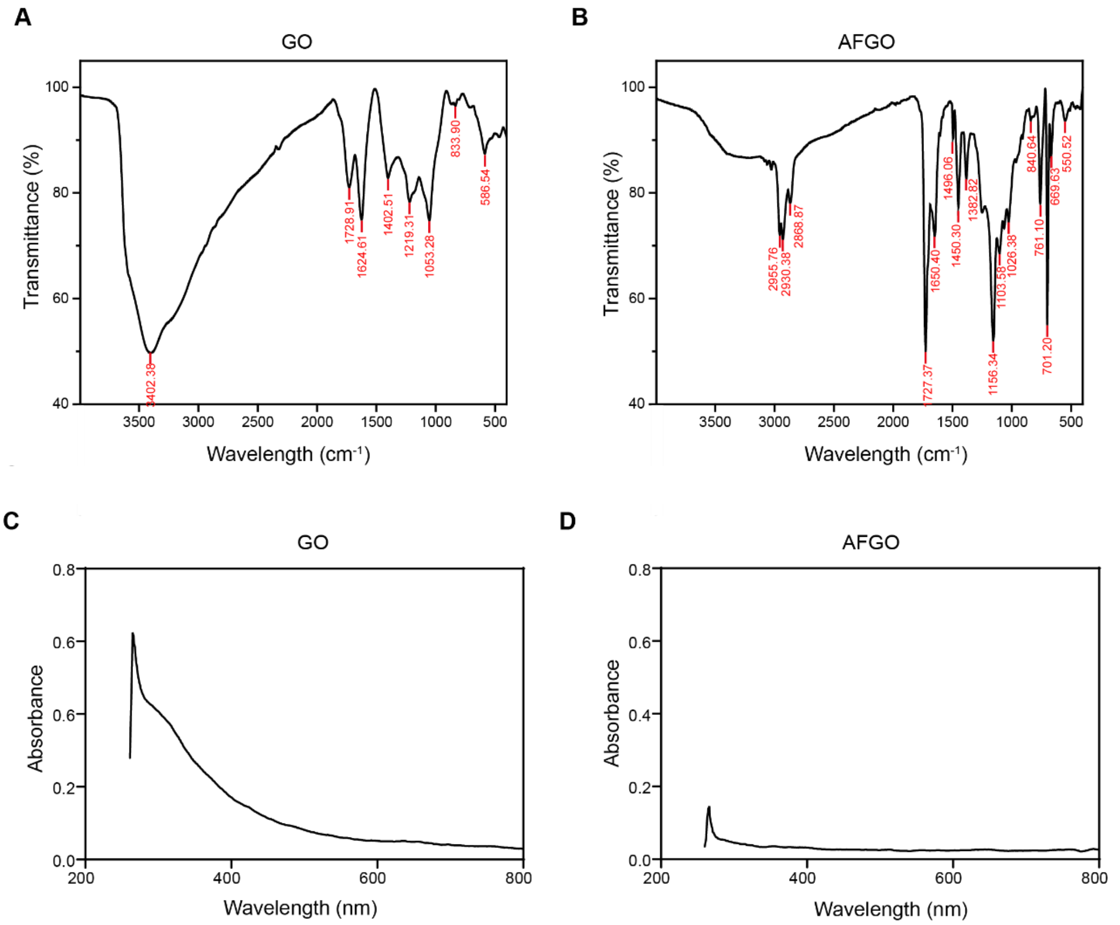

Figure S7. Characterization of the synthesized AFGO. Upper panel, the FT-IR spectra of GO and AFGO. The FT-IR spectra of GO showed several peaks such as the –OH group at 3402.38 cm^{-1}, –C=O group at 1,728.91 cm^{-1}, the aromatic C=C skeletal vibrations at 1624.61 cm^{-1}, and C–O–C/C–OH vibrations at 1,000–1,400 cm^{-1}, which were in accordance with the literature data.[1, 2] These results indicate that abundant oxygen-containing functional gro existists on GO. After functionalizing with acrylic acid, the OH group peak at 3402.38 cm−1 faded but the –C=O group peak at 1,728.91 cm^{-1} enhanced. Besides, the AFGO has a characteristic =C–H peak at 2955.76 cm^{-1} and a characteristic –C=C– peak at 840.64 cm^{-1}, indicating that a portion of –OH groups have converted into –O–CO–CH=CH_2 (Supplementary Figure S7 B). The characteristic UV-Vis spectrum peak of GO significantly declined after the modification (Supplementary Figure S7 C and D).

Figure S8. Dispersion performance of GO and AFGO in hydrogels

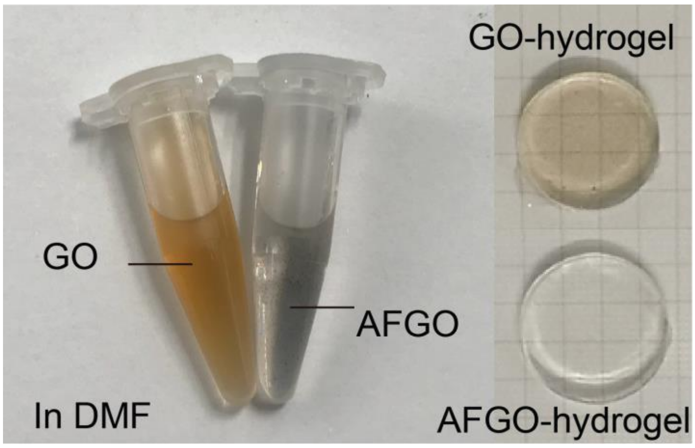

Figure S8. Dispersion performance of GO and AFGO in gels. Optical images of GO and AFGO dispersed in DMF and images of GO-hydrogels and AFGO-hydrogels.

Figure S9. Contraction and swelling performance of the ANP hydrogel

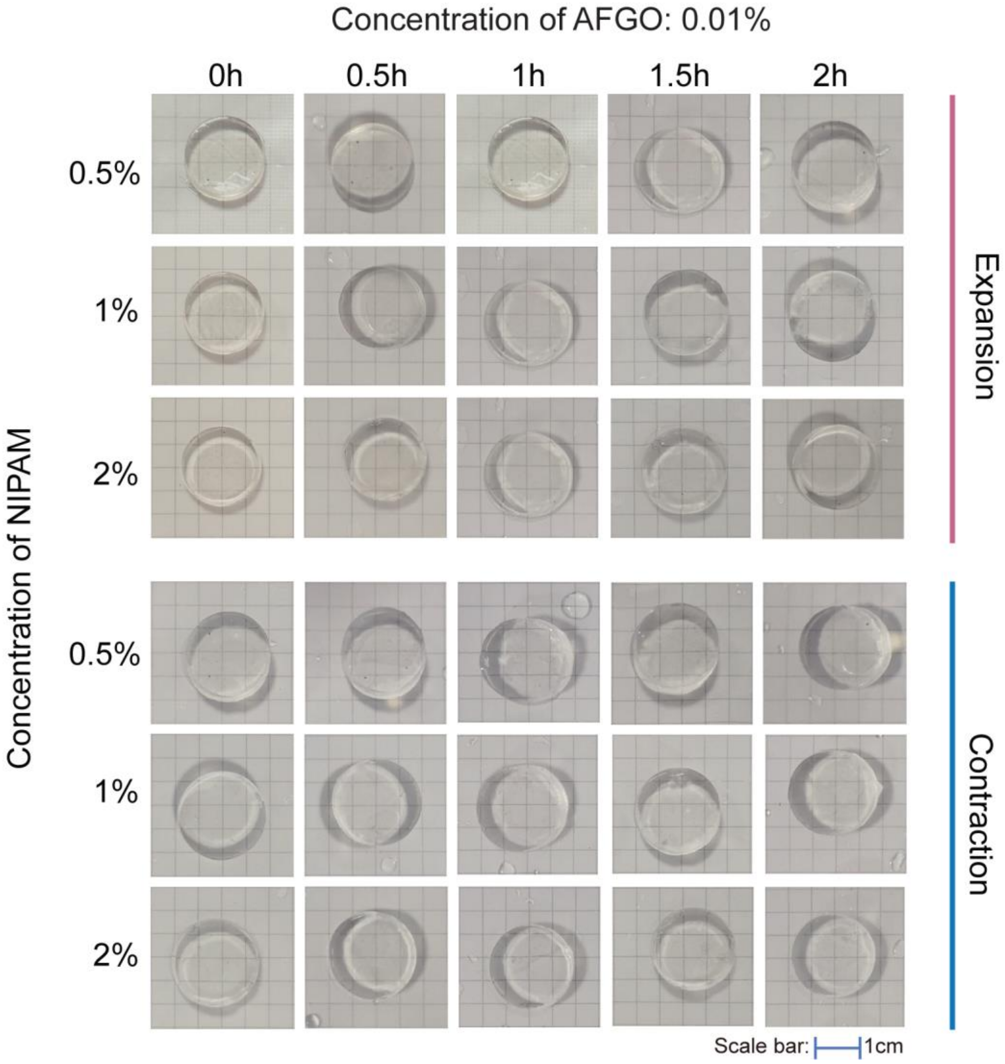

Figure S9. Contraction and swelling performance of the ANP hydrogel. Optical images of shrinkage and swelling properties of ANP hydrogels (0.5%, 1%, 2% NIPAM plus extra 0.01% AFGO) at 4 °C and 55 °C, continuous measurements from 0-2 hours at half-hour intervals.

Figure S10. Morphological characterization of the AFGO-hydrogels

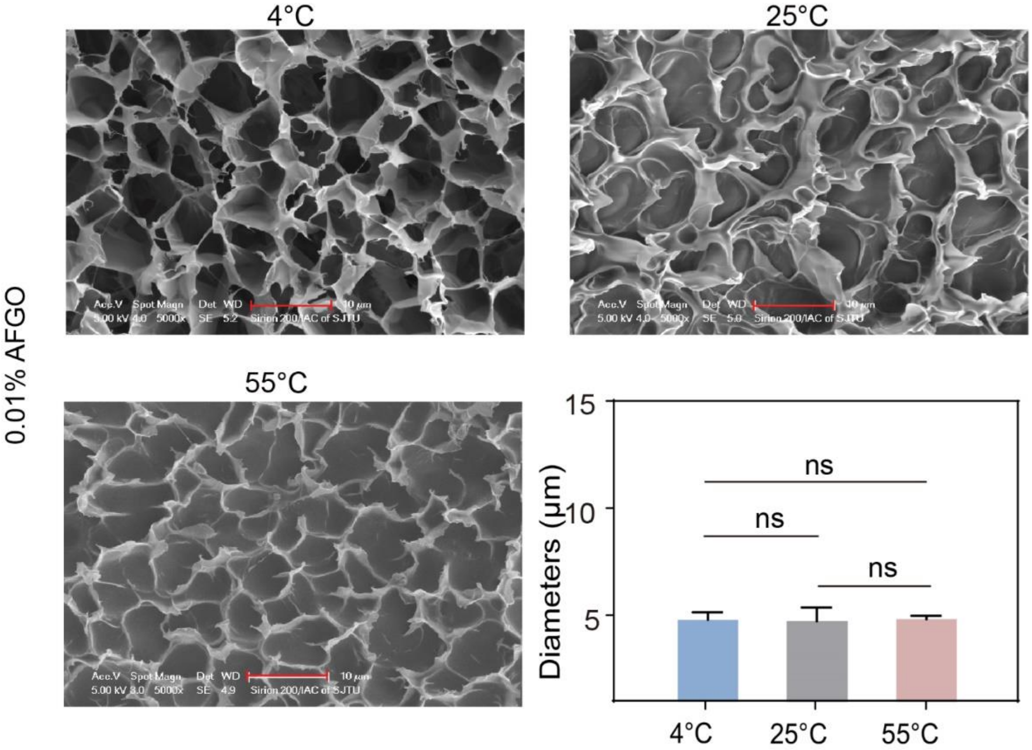

Figure S10. Morphological characterization of the AFGO-hydrogels (14T%, 0.01%AFGO). SEM images of porous structures in AFGO-hydrogels at 25 °C, 4 °C, and 55 °C. Scale bar: 10 μm. Histograms show the average pore size of gels at 25 °C, 4°,C and 55 °C. Data are expressed as mean ± SD. *P<0.05, **P<0.01, ***P<0.001, one-way ANOVA.

Figure S11. Comparison of proteins electrophoretic separation in non-ANP hydrogel (hydrogels without AFGO or NIPAM doping) and ANP hydrogel

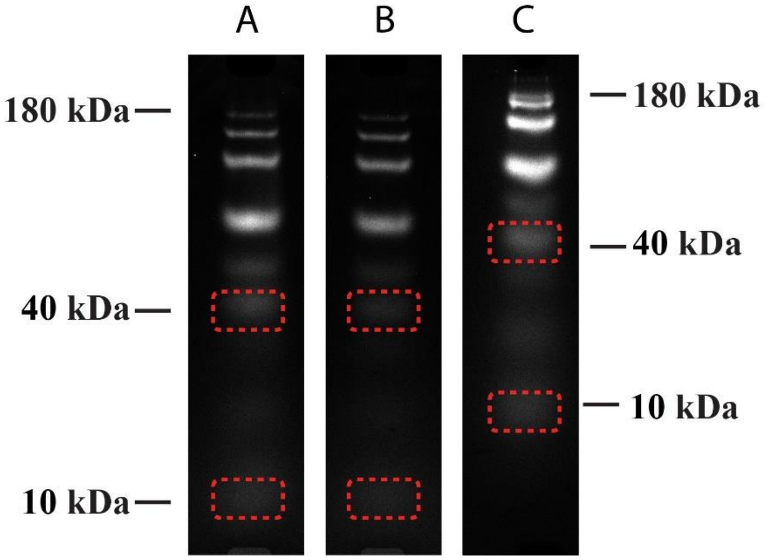

Figure S11. The separation performance of protein ladders (10kD - 180 kD) in the non-ANP hydrogel and ANP hydrogels. (A) The separation performance of protein ladders (10kD - 180 kD) in the non-ANP hydrogel at 25 °C electrophoresis time: 75s, electric field intensity: 40 V cm^{-1}, UV exposure: 60s. (B) The separation performance of protein ladders (10kD - 180 kD) in the ANP hydrogel at 25 °C. Electrophoresis time: 75s, electric field intensity: 40 V cm^{-1}, UV exposure: 60s. (C) The separation performance of protein ladders (10kD - 180 kD) in the ANP hydrogel at 55 °C. Electrophoresis time: 75s, electric field intensity: 40 V cm^{-1}, UV exposure: 60s

Figure S12. The photo capture protein performance of the ANP hydrogels

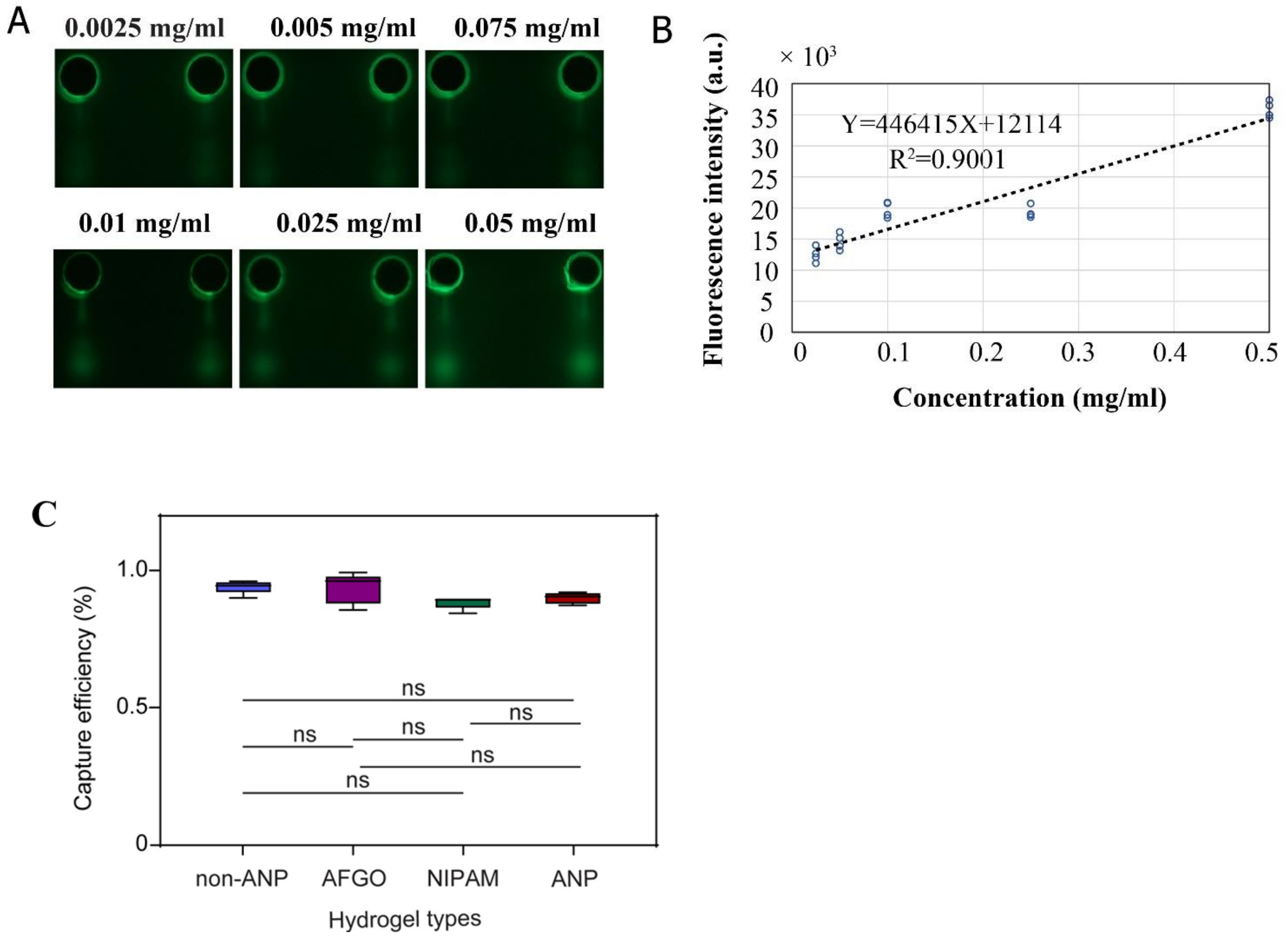

Figure S12. The photo capture protein performance of the ANP hydrogels. (A) the protein photo capture performance with fluorescein isothiocyanate labeled bovine serum albumin at different concentrations. Electrophoresis time: 30s, electric field intensity: 40 V cm^{-1}, UV exposure: 60s, number of parallel experiments: 6. (B) The quantitative linear relationship between the fluorescence intensity values of protein bands and the FITC-BSA concentrations. (C) Comparison of photo capture performance of the NIPAM-hydrogel, AFGO- hydrogel, ANP hydrogel, and the undoped non-ANP hydrogel. Loading protein is FITC-BSA. MAP-mPyTC concentration is 0.6 mM, NIPAM concentration is 1% and AFGO concentration is 0.01%. Data were shown as mean ± SD. N = 5 for each data point. *P<0.05, **P<0.01, ***P<0.001, one-way ANOVA.

Figure S13. The electrophoretic migration stability experiment.

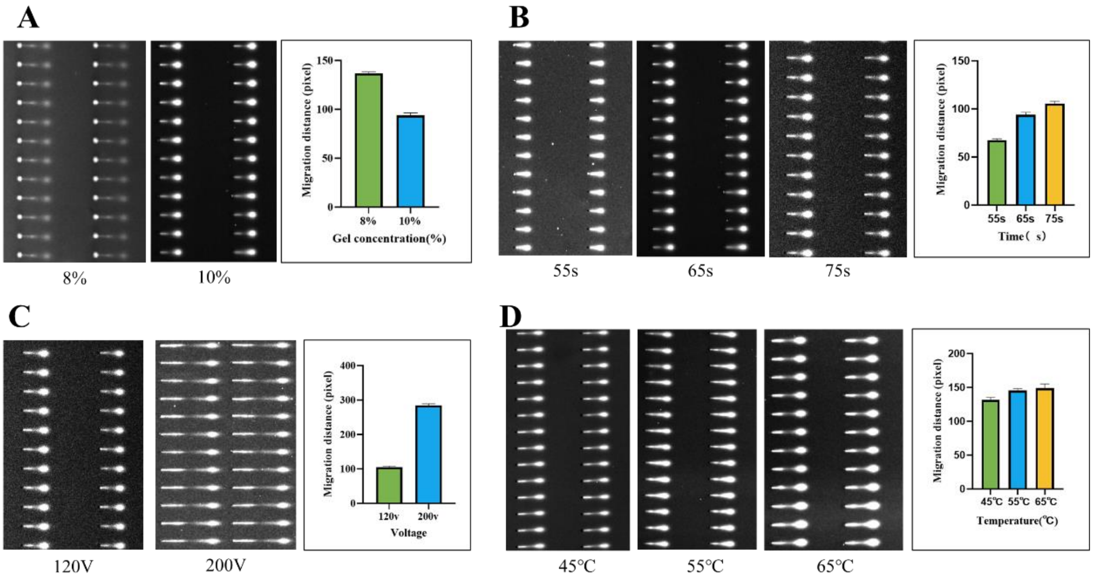

Figure S13. The electrophoretic migration stability experiment. (A) Electrophoretic migration behavior visual comparison and the corresponding quantitative data of electrophoretic distance in 8%T gel and 10%T gel. Electrophoresis time: 65s, voltage:120V. (B) Electrophoretic migration behavior visual comparison and the corresponding quantitative data of electrophoretic distance in different times. Voltage:120V, Data are expressed as mean ± SD. (C) Electrophoretic migration behavior visual comparison and the corresponding quantitative data of electrophoretic distance in different temperatures. Voltage:120V, electrophoresis time: 75s. (D) Electrophoretic migration behavior visual comparison and the corresponding quantitative data of electrophoretic distance in two different electric field intensities. Electrophoresis time: 75s.

Figure S14. Comparison of different antibody incubation methods

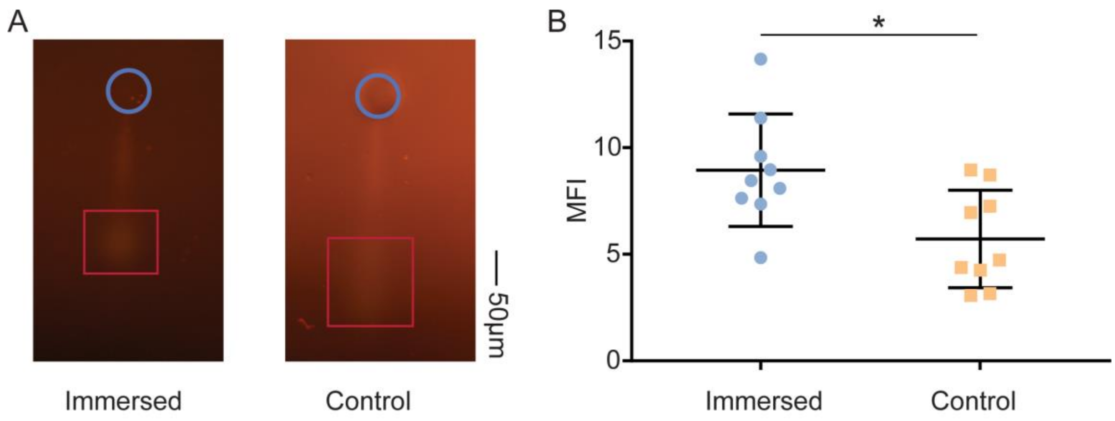

Figure S14. Comparison of different antibody incubation methods. (A) Visual images and (B) corresponding quantitative data of immunoblotting fluorescence intensity by immersed antibody incubation and surface-contact antibody incubation (control). Scale bar: 50 μm. Data were shown as mean ± SD. *P<0.05, **P<0.01, ***P<0.001, t test.

Figure S15. Comparing the diameters of microwells in 4 °C and 55 °C ANP gels

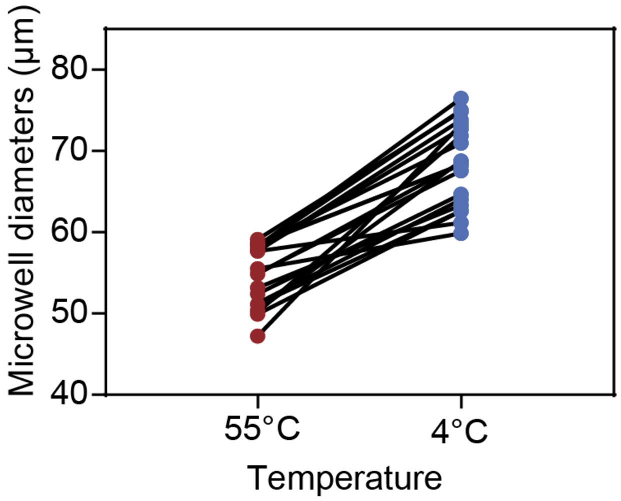

Figure S15. Comparing the diameters of microwells in 4 °C and 55 °C ANP gels. The original diameter of the microwell is 60 μm.

Figure S16. Gating strategy to discriminate single and live CHOP-positive cells.

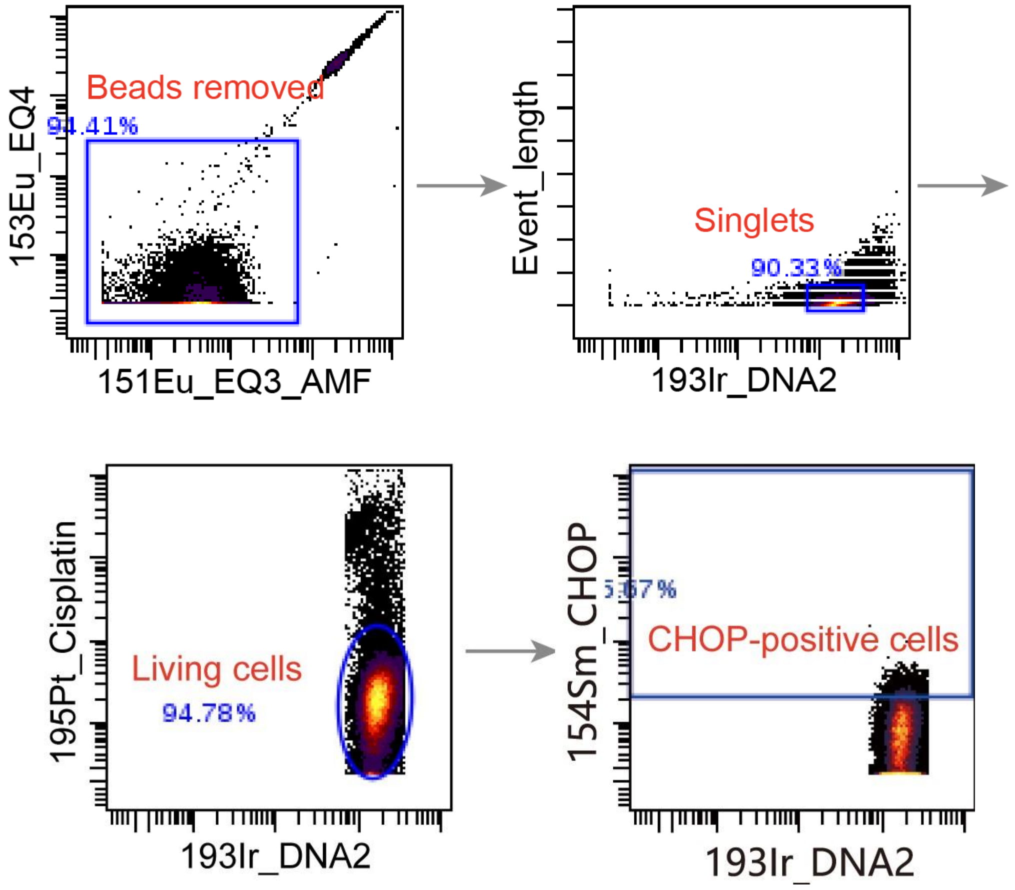

Figure S16. Gating strategy to discriminate single and live CHOP-positive cells. Different cell lines (BxPC-3, Panc-1, SW1990, and HPDE) were analyzed with mass cytometry.

Table S1. Preparation of N- isopropylacrylamide doped PA-gel

NIPAM%	Tris-HCl (pH8.8)	NIPAM (10%)	Acr (60%)	BIS 9.58 x	ddH_2O	5% SDS	5% Triton	10% TEMED	10% APS
1	250(μl)	500(μl)	750(μl)	523(μl)	2697(μl)	100(μl)	100(μl)	40(μl)	40(μl)
2	250(μl)	1000(μl)	667(μl)	523(μl)	2280(μl)	100(μl)	100(μl)	40(μl)	40(μl)
0.5	250(μl)	250(μl)	792(μl)	523(μl)	2905(μl)	100(μl)	100(μl)	40(μl)	40(μl)

Table S2 Rheological properties of the Non-ANP hydrogel and the ANP hydrogel

Frequency sweep of Non-ANP hydrogel (8%T)

Storage modulus	Loss modulus	Tan(delta)	Angular frequency	Oscillation torque	Step time	Temperature	Raw phase	Oscillation displacement	Complex viscosity
MPa	MPa		rad/s	μN.m	s	°C	°	rad	cP
0.01	0.00	-0.04	0.63	11.64	80.66	25.00	-2.40	0.01	18229900.00
0.01	0.00	0.21	1.00	11.10	106.61	25.00	12.01	0.01	11135200.00
0.01	0.00	-0.08	1.58	11.43	131.77	25.00	-4.64	0.01	7350120.00
0.01	0.00	-0.19	2.50	10.20	153.06	25.00	-11.46	0.01	4442110.00
0.01	0.00	-0.04	3.96	7.43	173.70	25.00	-2.63	0.01	2460770.00
0.01	0.00	-0.03	6.28	6.44	184.52	25.00	-3.05	0.01	1903500.00
0.01	0.00	-0.02	9.96	2.33	208.62	25.00	186.26	0.01	1186810.00
0.01	0.00	0.05	15.78	22.81	233.77	25.00	178.49	0.01	792774.00
0.01	0.00	0.04	25.01	75.96	252.31	25.00	179.64	0.01	495437.00
0.01	0.00	0.07	39.64	209.24	264.90	25.00	179.78	0.01	296350.00
0.01	0.00	0.06	62.83	543.67	277.81	25.00	179.93	0.01	172271.00
0.01	0.00	0.12	99.58	1391.01	290.53	25.00	179.93	0.01	138193.00
0.02	0.00	0.14	157.83	3502.08	303.22	25.00	179.96	0.01	117392.00
0.03	0.01	0.21	250.14	8805.11	316.03	25.00	179.97	0.01	104066.00
0.10	0.00	0.00	396.44	22089.40	328.97	25.00	180.00	0.01	247101.00
0.50	0.34	0.68	628.32	55293.20	341.76	25.00	179.65	0.01	962277.00

Amplitude sweep of Non-ANP hydrogel (8%T)

Storage modulus	Loss modulus	Tan(delta)	Angular frequency	Oscillation torque	Step time	Temperature	Raw phase	Oscillation displacement	Oscillation strain	Oscillation stress
MPa	MPa		rad/s	µN.m	s	°C	°	rad	%	MPa
0.012595	0.000431	0.034204	6.28319	0.7055	10.3898	25	3.49641	0.000609	0.099348	1.25E-05
0.011518	0.000568	0.049357	6.28319	1.0045	31.5675	25	5.43636	0.00102	0.166514	1.92E-05
0.011274	0.001468	0.130203	6.28319	1.6706	52.6592	25	14.3698	0.001721	0.280958	3.19E-05
0.010918	0.00219	0.200607	6.28319	2.5958	73.6995	25	22.1745	0.002725	0.444939	4.95E-05
0.010714	0.002839	0.26499	6.28319	3.9514	94.8736	25	28.7739	0.004082	0.666372	7.39E-05
0.010821	0.003029	0.279908	6.28319	6.3007	116.137	25	29.8559	0.006309	1.0301	0.000116
0.012614	0.003169	0.251245	6.28319	10.412	137.447	25	24.1463	0.008188	1.33689	0.000174
0.013041	0.001842	0.141242	6.28319	19.5741	158.71	25	13.804	0.015448	2.52217	0.000332
0.012295	0.00135	0.109812	6.28319	27.8376	179.849	25	11.3103	0.024638	4.02249	0.000498
0.01129	0.001502	0.133076	6.28319	38.2202	201.226	25	14.6535	0.039207	6.40117	0.000729
0.009962	0.002195	0.220365	6.28319	50.4694	222.434	25	26.4252	0.062338	10.1776	0.001038
0.008559	0.00288	0.336529	6.28319	66.6229	243.541	25	43.6972	0.097361	15.8957	0.001435
0.007805	0.003238	0.414929	6.28319	79.766	259.446	25	55.0861	0.12306	20.0914	0.001698

Frequency sweep of ANP hydrogel (8%T+1% NIPAM)

Storage modulus	Loss modulus	Tan(delta)	Angular frequency	Oscillation torque	Step time	Temperature	Raw phase	Oscillation displacement	Complex viscosity
MPa	MPa		rad/s	µN.m	s	°C	°	rad	cP
0.002007	0.000113	0.056262	0.628319	1.9892	60.5103	25	3.29429	0.005036	3198850
0.001718	0.000658	0.382768	0.995816	1.7305	86.759	25	22.288	0.004963	1847670
0.002033	-0.00023	-0.1112	1.57827	1.7454	111.959	25	-7.37186	0.004928	1295840
0.002165	0.00016	0.07408	2.50139	1.1348	133.356	25	6.32225	0.003876	867750
0.001892	2.44E-05	0.012889	3.96441	0.0984	153.886	25	15.1397	0.005239	477269
0.002116	0.000132	0.062399	6.28319	2.4383	175.071	25	176.865	0.005024	337350
0.00228	-1.4E-05	-0.00594	9.95809	9.1898	187.273	25	180.085	0.005029	228931
0.002405	9.46E-05	0.039337	15.7828	26.2958	206.294	25	179.793	0.005001	152471
0.002513	0.000137	0.054331	25.0137	69.2485	218.621	25	179.887	0.004976	100604
0.002633	0.000181	0.068672	39.6438	178.444	231.229	25	179.942	0.004999	66560.1
0.002462	0.000258	0.104646	62.8319	453.025	244.227	25	179.967	0.005005	39397.8
0.003616	0.000504	0.13949	99.5816	1140.58	257.179	25	179.975	0.005006	36659.8
0.00602	0.001217	0.202083	157.827	2868.04	270.194	25	179.976	0.005006	38915.7
0.008235	0.004069	0.494022	250.14	7201.64	283.098	25	179.967	0.004999	36721.9
0.061371	-0.00264	-0.04307	396.441	18056.7	295.941	25	180.008	0.005001	154949
0.401906	0.266844	0.663947	628.319	45149.1	308.658	25	179.659	0.005006	767803

Amplitude sweep of ANP hydrogel (8%T+1% NIPAM)

Storage modulus	Loss modulus	Tan(delta)	Angular frequency	Oscillation torque	Step time	Temperature	Raw phase	Oscillation displacement	Oscillation strain	Oscillation stress
MPa	MPa		rad/s	µN.m	s	°C	°	rad	%	MPa
0.002219	9.59E-05	0.043216	6.28319	0.2318	20.8261	25	177.62	0.000499	0.09987	2.22E-06
0.002207	0.000148	0.067254	6.28319	0.3701	36.9648	25	176.336	0.000792	0.158463	3.51E-06
0.002168	0.000137	0.063265	6.28319	0.5762	58.2594	25	176.672	0.001213	0.242647	5.27E-06
0.002205	0.000126	0.057046	6.28319	0.9279	74.1171	25	176.898	0.001985	0.397073	8.77E-06
0.002184	0.000137	0.062674	6.28319	1.4825	90.0547	25	176.656	0.003143	0.628542	1.38E-05
0.002197	0.000111	0.050395	6.28319	2.3628	106.115	25	177.278	0.00504	1.00802	2.22E-05
0.002167	0.000136	0.062563	6.28319	3.7527	122.051	25	176.71	0.0079	1.57995	3.43E-05
0.002161	0.000128	0.05914	6.28319	6.0351	138.191	25	176.907	0.012673	2.53457	5.49E-05
0.002115	0.000141	0.066615	6.28319	9.6392	154.22	25	176.655	0.019851	3.97026	8.42E-05
0.002061	0.000155	0.075309	6.28319	15.8621	170.406	25	176.398	0.03194	6.38788	0.000132
0.001953	0.000195	0.099705	6.28319	26.0125	191.716	25	175.67	0.050147	10.0295	0.000197
0.001849	0.000255	0.138184	6.28319	42.5872	207.59	25	174.549	0.078758	15.7517	0.000294
0.001728	0.000303	0.175116	6.28319	56.178	223.65	25	173.828	0.099278	19.8562	0.000348

Frequency sweep of ANP hydrogel (10%T+1% NIPAM)

Storage modulus	Loss modulus	Tan(delta)	Angular frequency	Oscillation torque	Step time	Temperature	Raw phase	Oscillation displacement	Complex viscosity
MPa	MPa		rad/s	µN.m	s	°C	°	rad	cP
0.004307	0.000447	0.103744	0.628319	4.349	60.6341	25	5.99799	0.00607	6891400
0.004289	0.001028	0.239661	0.995816	4.2984	73.8227	25	13.9032	0.005997	4428770
0.004406	0.000394	0.08952	2.50139	3.9656	128.442	25	6.34843	0.006635	1768350
0.00413	0.000498	0.120485	6.28319	0.4198	169.844	25	159.069	0.001798	662089
0.004436	0.000165	0.037144	9.95809	9.1258	187.826	25	178.975	0.005915	445816
0.004866	0.000121	0.024903	15.7828	29.3671	200.794	25	179.764	0.005961	308407
0.005269	6.34E-05	0.012039	25.0137	81.5421	219.463	25	179.955	0.006022	210676
0.005455	0.000215	0.039488	39.6438	211.759	232.071	25	179.941	0.005997	137703
0.00621	0.000212	0.034208	62.8319	540.537	244.929	25	179.977	0.006009	98898.3
0.006712	0.000794	0.118267	99.5816	1364.24	257.787	25	179.966	0.005998	67872
0.011186	0.002433	0.217505	157.827	3434.67	270.77	25	179.959	0.006001	72532.9
0.014189	0.006027	0.424786	250.14	8627.55	283.738	25	179.96	0.005992	61628.4
0.069453	-0.004149	-0.059734	396.441	21667.8	296.768	25	180.011	0.006	175503
0.31746	0.544928	1.71652	628.319	54422.1	309.625	25	179.422	0.006015	1003720

Amplitude sweep of ANP hydrogel (10%T+1% NIPAM)

Storage modulus	Loss modulus	Tan(delta)	Angular frequency	Oscillation torque	Step time	Temperature	Raw phase	Oscillation displacement	Oscillation strain	Oscillation stress
MPa	MPa		rad/s	μN.m	s	°C	°	rad	%	MPa
0.005079	0.000524	0.103259	6.28319	0.0987	5.15569	25	123.901	0.000932	0.155345	7.93E-06
0.005557	0.000552	0.099393	6.28319	0.0983	10.7801	25	77.1919	0.001035	0.172561	9.64E-06
0.005338	0.000132	0.024663	6.28319	0.0949	32.0674	25	125.32	0.00351	0.584945	3.12E-05
0.005328	0.000209	0.039248	6.28319	0.1603	53.5182	25	116.271	0.004102	0.683715	3.65E-05
0.005204	9.63E-05	0.018509	6.28319	0.3207	74.7476	25	157.005	0.007763	1.29391	6.73E-05
0.005212	0.000188	0.036027	6.28319	0.2905	90.8707	25	139.48	0.006	1.00003	5.22E-05
0.005032	0.000129	0.025546	6.28319	1.0308	133.147	25	162.167	0.014657	2.44289	0.000123
0.004944	0.00024	0.048627	6.28319	2.2853	154.239	25	153.75	0.025094	4.18236	0.000207
0.004696	0.000271	0.057645	6.28319	4.8757	175.299	25	159.788	0.037141	6.19019	0.000291
0.004467	0.000345	0.077155	6.28319	9.9313	196.5	25	160.322	0.057907	9.65131	0.000432
0.004136	0.000529	0.127928	6.28319	21.6006	217.763	25	157.78	0.09214	15.3567	0.00064
0.003943	0.000661	0.167663	6.28319	31.8686	238.995	25	156.049	0.116797	19.4666	0.000778

Frequency sweep of ANP hydrogel (8%T+2% NIPAM)

Storage modulus	Loss modulus	Tan(delta)	Angular frequency	Oscillation torque	Step time	Temperature	Raw phase	Oscillation displacement	Complex viscosity
Pa	Pa		rad/s	µN.m	s	°C	°	rad	Pa.s
8613.84	199.53	0.023164	0.628319	4.31774	12.9813	25	1.33157	0.001688	13713
8644.08	201.866	0.023353	0.995816	4.30445	22.8432	25	1.34951	0.001686	8682.77
8686.47	179.757	0.020694	1.57827	4.268	30.5149	25	1.21182	0.001686	5504.96
8722.75	174.016	0.01995	2.50139	4.14466	36.7782	25	1.20852	0.001686	3487.85
8770.47	174.709	0.01992	3.96441	3.80809	43.7121	25	1.32038	0.001686	2212.74
8800.36	174.586	0.019839	6.28319	2.92216	50.2952	25	1.72124	0.001688	1400.9
8842.47	176.743	0.019988	9.95809	0.674956	57.0882	25	7.56635	0.001688	888.146
8925.59	184.576	0.02068	15.7828	5.00189	63.9971	25	178.937	0.001688	565.647
9062.36	191.331	0.021113	25.0137	19.3293	70.5864	25	179.714	0.001693	362.376
9358.4	189.344	0.020233	39.6438	55.4637	77.2494	25	179.901	0.001697	236.11
10169.4	47.9834	0.004718	62.8319	145.886	84.0923	25	179.99	0.001695	161.853
11374.3	-435.744	-0.03831	99.5816	372.64	90.8222	25	180.034	0.001691	114.305
12740.2	-2264.9	-0.177776	157.827	942.768	97.3561	25	180.069	0.001689	81.988
22597.3	-8286.18	-0.366689	250.14	2375.96	103.889	25	180.101	0.001691	96.2206
58366	-33173.2	-0.568366	396.441	5972.51	110.586	25	180.16	0.001692	169.343
213977	-193305	-0.903392	628.319	15000.2	117.417	25	180.373	0.001696	458.944

Amplitude sweep of ANP hydrogel (8%T+2% NIPAM)

Storage modulus	Loss modulus	Tan(delta)	Angular frequency	Oscillation torque	Step time	Temperature	Raw phase	Oscillation displacement	Oscillation strain	Oscillation stress
Pa	Pa		rad/s	µN.m	s	°C	°	rad	%	Pa
9407.72	165.533	0.017596	6.28319	0.646266	5.87604	25	1.47765	0.000338	0.100145	9.42282
9397.39	181.489	0.019313	6.28319	1.02124	12.4389	25	1.62262	0.000535	0.158495	14.8972
9382.79	160.5	0.017106	6.28319	1.61543	19.0157	25	1.43832	0.000848	0.251307	23.583
9375.63	175.023	0.018668	6.28319	2.55616	25.55	25	1.57018	0.001344	0.398077	37.3288
9366.42	177.903	0.018994	6.28319	4.04618	32.2109	25	1.59829	0.00213	0.631015	59.1142
9333.24	195.74	0.020972	6.28319	6.38385	39.0638	25	1.76764	0.003377	1.00071	93.4192
9284.07	225.741	0.024315	6.28319	10.0442	45.8407	25	2.05426	0.005354	1.58652	147.337
9187.46	282.947	0.030797	6.28319	15.6997	52.4078	25	2.6143	0.008496	2.5175	231.404
9001.47	394.064	0.043778	6.28319	24.2101	59.2377	25	3.7509	0.013493	3.99792	360.216
8653.72	630.47	0.072855	6.28319	36.4169	66.0346	25	6.35255	0.021456	6.35733	551.604
8008.95	1115.86	0.139326	6.28319	52.3668	72.8261	25	12.536	0.034196	10.1325	819.342
7176.11	1973.79	0.27505	6.28319	74.7518	79.6448	25	25.2473	0.054229	16.0682	1195.9
6723.11	2392.27	0.355828	6.28319	89.8123	86.4244	25	32.6569	0.06801	20.1514	1438.01

Table S3. Preparation of ANP hydrogel

NIPAM%	Tris-HCl (pH8.8)	NIPAM (10%)	Acr (60%)	BIS 9.58x	ddH_2O	5% SDS	5% Triton	0.01% AFGO	10% TEMED	10% APS
1	250(μl)	500(μl)	750(μl)	523(μl)	2647(μl)	100(μl)	100(μl)	50(μl)	40(μl)	40(μl)
2	250(μl)	1000(μl)	667(μl)	523(μl)	2230(μl)	100(μl)	100(μl)	50(μl)	40(μl)	40(μl)
0.5	250(μl)	250(μl)	792(μl)	523(μl)	2855(μl)	100(μl)	100(μl)	50(μl)	40(μl)	40(μl)

Table S4. Antibodies used for mass cytometry

Antibody	Manufacturer	Item No.	Clone	Mass tag	Species	Molecular weight
CHOP	ThermoFisher	PA5-35129	Polyclonal	^{154}Sm	Rabbit	19 kDa
MDH2	Abcam	ab135530	Polyclonal	^{170}Er	Rabbit	36 kDa
GAPDH	Abcam	ab9484	Polyclonal	^{171}Yb	Mouse	37 kDa
FH	Abcam	ab234907	EPR21105	^{173}Yb	Rabbit	54 kDa

Chemicals and materials

Chemicals

TWEEN-20 (P1379), Triton X-100 (T8787), and ammonium persulfate (APS, A3678) were obtained from Sigma-Aldrich. Protein markers were purchased from Yeasen Biotech. Tetramethylethylenediamine (TEMED, TB0508), acrylamide/bis-acrylamide 30% solution (29:1) (B546017), acrylamide and N, N′-Methylenebisacrylamide were purchased from Energy Chemical, 10× TBST buffer and other conventional western blot associated chemicals and buffer were purchased from Sangon Biotech. Deionized water (18.2 MΩ) was obtained via an ultrapure water system from Millipore. N-(3-methacrylamidopropyl)-2-(1-methyl-1H-pyrrol-2-yl)-2H-tetrazole-5-carboxamide (MAP-mPyTC) were synthesized in-house as previously described. N-isopropylacrylamide (NIPAM) was obtained from shyuanye (S30716). Acrylic acid, dichlorosulfoxide, hydroquinone and triethylamine were obtained from Aladdin. Tunicamycin was obtained from Abcam (ab120296-10mg). 500 ml lysis/electrophoresis buffer was prepared in house (25 ml 10x Tris-HCL buffer, 2.5 g SDS, 1.25 g sodium deoxycholate, 500 μL TritonX-100, 474.5 ml ddH_2O). Dissolve and mix the buffer thoroughly before use. Store at 4°C.

Protein standards and antibodies

Protein standards include BSA (66 kDa, Sangon Biotech), Trypsin inhibitor (TI) (20 kDa, Sangon Biotech), FITC-ConA (MP6321-5MG, MKBio), FITC-OVA (SF069, Solarbio), FITC-BSA (SF063, Solarbio), FITC-lysozyme (R-FL-001, RuixiBio). Antibodies employed contain anti-bovine serum albumin [EPR12774] (ab192603, Abcam), rabbit polyclonal to Trypsin Inhibitor antibody (ab34549, Abcam), anti-MDH2 antibody (ab135530, Abcam), anti-FH/Fumarase antibody (ab234907, Abcam), anti-GAPDH antibody (ab9484, Abcam), anti-CHOP polyclonal antibody (PA5-35129, Thermo Fisher), anti-β-Tubulin (O95C1, Biolegend), Alexa Fluor 488 AffiniPure Goat anti- Mouse IgG (H+L) (33206ES60, Yeasen Biotech), and Alexa Fluor 594 AffiniPure Goat Anti-Rabbit IgG (H+L) (33112ES60, Yeasen Biotech).

Methods

Temperature control strategies for tc-scWB

First, the microchip and the electrophoresis solution were preheated to 55°C before electrophoresis with a water bath. After 10 s of cell lysis process, start electrophoresis at a field strength of 30-40 V/cm.

Electrophoresis time varies (30-70 s) based on the molecular weight of the target protein. For the molecular weights in the range of 20-100 kDa, the electrophoresis time of 45- 75s is preferred. The temperatures of the buffer would range smoothly under the selected electrophoresis time. Once electrophoresis and protein fixation were completed, the slides were placed in custom-sized film bags on a shaking plate and incubated overnight at 4°C, the antibody staining and blotting steps were compatible with the traditional Western Blot. After incubation was completed, the microchip was then incubated at 55°C in a water bath to further concentrate the fluorescence signal.

References

1. D. W. Lee *et al.*, The Structure of Graphite Oxide: Investigation of Its Surface Chemical Groups. *The Journal of Physical Chemistry B* **114**, 5723-5728 (2010).
2. Y. Matsuo, T. Tabata, T. Fukunaga, T. Fukutsuka, Y. Sugie, Preparation and characterization of silylated graphite oxide. *Carbon* **43**, 2875-2882 (2005).
